# Application of telemedicine in fatigue management for patients with multiple sclerosis: A scoping review

**DOI:** 10.1371/journal.pone.0327563

**Published:** 2025-07-17

**Authors:** Xiaoyan Gong, Xiaoyu Xue, Rong Gao, Shengya Feng, Xinyu Ji, Jie Zheng, Bowen Xue

**Affiliations:** 1 School of Nursing, Shanxi Medical University, Taiyuan, Shanxi, China; 2 Academy of Medical Sciences, Shanxi Medical University, Taiyuan, Shanxi, China; 3 Shanxi Bethune Hospital, Taiyuan, Shanxi, China; 4 Affiliated Mental Health Center & Hangzhou Seventh People’s Hospital, Zhejiang University School of Medicine, Hangzhou, Zhejiang, China; 5 Center for Humanistic Psychiatry, Zhejiang University School of Medicine, Hangzhou, Zhejiang, China; University of Campania Luigi Vanvitelli: Universita degli Studi della Campania Luigi Vanvitelli, ITALY

## Abstract

**Background:**

Fatigue is a prevalent symptom in people with Multiple Sclerosis, but evidence for the effectiveness of telemedicine in treating this symptom remains incomplete. Despite favorable clinical trial results, its integration into practice and systematic evaluation is limited.

**Objective:**

The purpose of this research project is to carefully assess how well telemedicine works for managing fatigue in MS patients.

**Methods:**

This scoping review adhered to the Joanna Briggs Institute methodological framework and followed the preferred reporting items for systematic reviews and meta-Analyses extension for ccoping reviews (PRISMA-ScR) guidelines. reporting guidelines. A search covering literature in both English and Chinese up until December 2024 was carried out in the electronic databases of PubMed, Embase, Web of Science, CINAHL, Cochrane Library, China National Knowledge Infrastructure (CNKI), Wan Fang, and VIP database. Studies that assessed telemedicine-based therapies for patients with multiple sclerosis and documented fatigue-related outcomes were eligible. The collected literature was compiled, examined, and pertinent information was extracted by two independent reviewers.

**Results:**

A total of 26 papers were included, all in English. Applications(n = 11), wearable devices(n = 8), teleconferences(n = 11), online platforms(n = 5), text messaging(n = 1), virtual reality(n = 1), and game consoles(n = 1) are some of the intervention forms of telemedicine. Remote monitoring(100% of studies), remote guidance(54%), and remote rehabilitation(58%) are some of the functional characteristics of telemedicine. Fatigue characteristics and its impact, health-related quality of life, physical activity, mental health, and the feasibility of remote interventions are among the outcome indicators. While 77% of studies reported statistically significant fatigue reduction, effect sizes varied from small to moderate.

**Conclusion:**

Telemedicine demonstrates potential as a viable alternative to conventional rehabilitation for managing MS-related fatigue, particularly through multimodal interventions enabling personalized and real-time management. However, the heterogeneity in influencing factors and treatment effects warrants validation through large-scale trials. Future research should prioritize multimodal strategies, optimizing sample composition, extending follow-up periods, and integrating standardized assessment tools to enhance intervention precision.

## Introduction

Multiple sclerosis (MS), a chronic autoimmune disease of the central nervous system, represents the leading cause of non-traumatic neurological disability in young adults worldwide [1]. Characterized by inflammatory demyelination and axonal damage, this condition affects over 2.8 million individuals globally, with peak diagnosis occurring around age 32 [2]. While its exact etiology remains elusive, emerging evidence suggests multifactorial interactions between viral exposures, environmental triggers, genetic predisposition, and lifestyle factors [2]. Current treatment guidelines emphasize the critical role of patient engagement in shared decision-making processes to optimize therapeutic outcomes [3,4]. According to the Multiple Sclerosis Treatment Consensus Group (MSTCG), the aim of MS treatment is to maximize results by enhancing patients’ quality of life and limiting the disease’s progression as much as feasible [1].

Among the range of symptoms of MS, fatigue is one of the most prevalent and disabling, affecting 83% of patients and manifesting itself in the form of perceptible exhaustion and objectively measurable declines in performance [5]. A veteran survey shows that fatigue has multifaceted impacts, impairing cognitive function, emotional well-being, and daily activities [6]. Patients, who often spend substantial time and energy managing fatigue, express strong interest in personalized remote interventions tailored to their specific needs. Current management strategies-including medications, exercise programs, and cognitive behavioral therapy (CBT) – show limited efficacy and variable safety [2,3]. The diagnosis of MS is largely based on a study of clinical history, and early progressive deterioration is often asymptomatic [7,8]. Therefore, its fatigue management is in urgent need of innovative approaches.

In this context, telemedicine has emerged as a key innovation in the management of chronic diseases.Defined by the World Health Organization (WHO) as“*the field of knowledge and practice associated with the development and use of digital technologies to improve health*” [9,10], This definition incorporates the phrases “digital health” and “m-health”, covering several facets of health information systems, telemedicine, and e-health [11]. Telemedicine is a key innovation in healthcare that relies on information technology and connectivity to make health information sharing and medical services more convenient and to facilitate efficient collaboration between patients, doctors and healthcare professionals. It can effectively improve the safety, effectiveness and quality of healthcare while reducing healthcare cost [11–14].Tele-digital solutions, such as smartphone-based apps, wearables, and decision support algorithms, are increasingly being used in clinical trials and integrated into routine health care, and show great potential in home care for MS-related fatigue [7,15–17]. Telehealth CBT for MS-related fatigue has emerged as an acceptable and effective treatment [6].

However, despite the increasing number of studies on telemedicine in recent years, there is no consensus on its effectiveness in fatigue management. In particular, the heterogeneity of study designs, intervention formats, evaluation tools, and study populations in existing studies is high, making it difficult to synthesize and compare study results. In addition, there are fewer studies related to telemedicine in critically ill, elderly, and low-education patients, which may limit the widespread use of telemedicine in clinical practice. By means of a scoping review, this article aims to comprehensively analyze the evidence of existing studies, explore the effectiveness of telemedicine in MS fatigue management and its influencing factors, and provide a reference for future research and clinical practice.

## Methods

### Type of review

This study adopts a scoping review methodology to systematically map the application landscape of telemedicine in fatigue management for patients with multiple sclerosis. Unlike systematic reviews that focus on quantitative analysis of intervention efficacy, this investigation prioritizes three core dimensions: telemedicine intervention modalities, functional characteristics, and outcome measurement approaches, specifically addressing exploratory questions of “how” interventions are implemented and “what” specific measures are employed.The selection of scoping review methodology is justified by three principal considerations. First, while systematic reviews require stringent inclusion criteria and homogeneous data [18], the current evidence base demonstrates substantial heterogeneity, manifested through multimodal intervention designs (with the majority of studies adopting composite interventions), non-standardized assessment tools, and heterogeneous participant characteristics – factors that preclude conventional meta-analytic approaches. Second, the study objectives emphasize knowledge mapping rather than efficacy verification, necessitating systematic delineation of key concepts, evidence typologies, and research gaps within this domain. Finally, scoping review methodology offers distinct advantages for integrating evidence in complex clinical contexts, permitting the inclusion of diverse evidence types and enabling conceptual mapping – features that align optimally with the exploratory nature of this inquiry [19].

**Fig 1:**
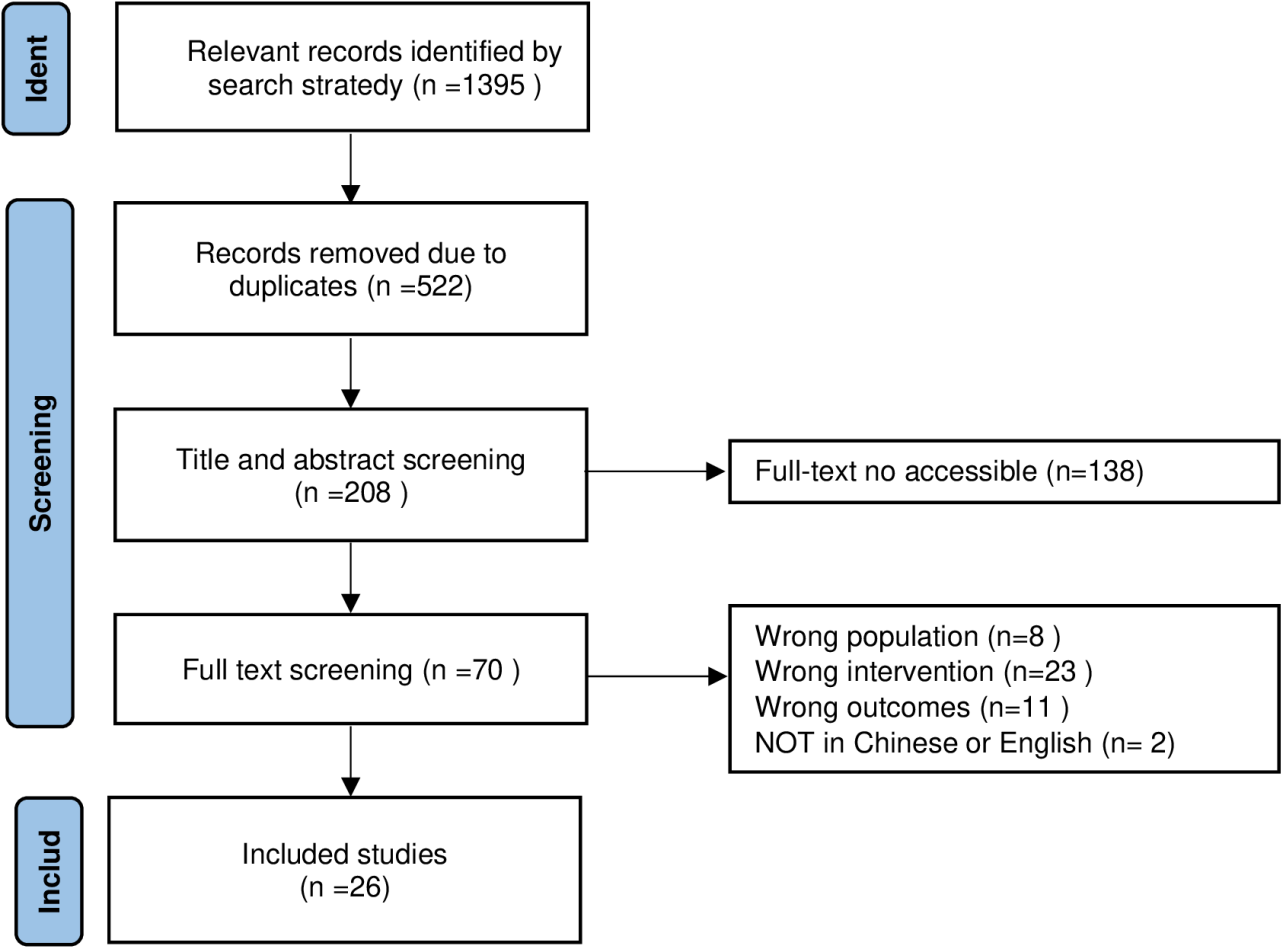
PRISMA flow chart of the selection process.

This study was conducted according to the Joanna Briggs Institute methodology for scoping reviews [20]. Reporting adhered to the preferred reporting items for systematic reviews and meta-analyses extension for scoping reviews (PRISMA-ScR) (Fig 1) [21].

### Identifying the research question

The specific research questions that guided this review were as follows: (i) what are the forms of intervention of telemedicine in fatigue management of MS patients? (ii) what are the functional characteristics of telemedicine in fatigue management of MS patients? (iii) what are the outcome measures of telemedicine in fatigue management of MS patients? (iv) what are the intervention effects of telemedicine in fatigue management of MS patients?

### Search strategy

A search was conducted in the electronic databases PubMed, Embase, Web of Science, CINAHL, Cochrane Library, CNKI, Wan Fang, and VIP database, covering literature in both English and Chinese up to December 2024. Common search fields were used, employing a combination of subject headings and free-text keywords. References were also tracked throughout the review process. The full search strategy is provided in Table 1 (S1 Table).

**Table 1:** Search strategy used for each of the databases.

PubMed	
#1	“Multiple Sclerosis”[Mesh]
#2	“Sclerosis”[Title/Abstract] OR “MS”[Title/Abstract] OR “Disseminated Sclerosis”[Title/Abstract])
#3	#1 OR #2
#4	“Fatigue”[Mesh]
#5	“Frailty”[Title/Abstract] OR “Asthenia”[Title/Abstract]OR “Muscle Weakness”[Title/Abstract]
#6	#4 OR #5
#7	“telemedicine”[Mesh]
#8	“Telehealth”[Title/Abstract] OR “Tele-Referral”[Title/Abstract] OR “Tele-Referrals” [Title/Abstract] OR “Mobile Health”[Title/Abstract] OR “mHealth”[Title/Abstract] OR “eHealth”[Title/Abstract] OR “Telecare”[Title/Abstract] OR “Digital Health” OR “App” [Title/Abstract] OR “Client-to-provider telemedicine”[Title/Abstract] OR “Digital biomarkers”[Title/Abstract] OR “Digital therapeutics”[Title/Abstract] OR “mobile terminal”[Title/Abstract] OR “smartphone”[Title/Abstract] OR “mobile application”[Title/Abstract] OR “smart application”[Title/Abstract] OR “wearable”[Title/Abstract] OR “smartwatch”[Title/Abstract] OR “Virtual Medicine”[Title/Abstract]
#9	#7 OR #8
#10	#3 AND #6 AND #9
Web of Science	
#1	TS=(“Multiple Sclerosis” OR “Sclerosis” OR “MS” OR “Disseminated Sclerosis”)
#2	TS=(“Fatigue” OR “Asthenia” OR” Frailty” OR” Muscle Weakness”)
#3	TS=(“telemedicine” OR “Telehealth” OR “Tele-Referral” OR “Tele-Referrals” OR “Mobile Health” OR “mHealth” OR “eHealth” OR “Telecare” OR “Digital Health” OR “App” OR “Client-to-provider telemedicine” OR “Digital biomarkers” OR “Digital therapeutics” OR “mobile terminal” OR “smartphone” OR “mobile application” OR “smart application” OR “wearable” OR “smartwatch” OR “Virtual Medicine”)
#4	#1 AND #2 AND #3
Cochrane	
#1	MeSH descriptor: [Multiple Sclerosis] explode all trees
#2	(Multiple Sclerosis or Sclerosis or MS orDisseminated Sclerosis):ti,ab,kw
#3	#1OR#2
#4	MeSH descriptor: [Asthenia] explode all trees
#5	(Frailty or Fatigue or Muscle Weakness):ti,ab,kw
#6	#4OR#5
#7	#3AND#6
#8	MeSH descriptor: [Telemedicine] explode all trees
#9	(Telehealth or Tele-Referral or Tele-Referrals or Mobile Health or mHealth or eHealth or Telecare or Digital Health or App or Client-to-provider telemedicine or Digital biomarkers or Digital therapeutics or mobile terminal or smartphone or mobile application or smart application or wearable or smartwatch or Virtual Medicine):ti,ab,kw
#10	#8OR#9
#11	#7AND#10
Embase	
#1	‘Multiple Sclerosis’/exp
#2	‘Sclerosis’:ti,ab,kw OR ‘MS’:ti,ab,kw OR ‘Disseminated Sclerosis’:ti,ab,kw
#3	#1 OR #2
#4	‘Fatigue’/exp
#5	Asthenia:ti,ab,kw OR Frailty:ti,ab,kw OR Muscle Weakness:ti,ab,kw
#6	#4 OR #5
#7	‘telemedicine’/exp
#8	Telehealth:ti,ab,kw OR Tele-Referral:ti,ab,kw OR Tele-Referrals:ti,ab,kw OR Mobile Health:ti,ab,kw OR mHealth:ti,ab,kw OR eHealth OR Telecare:ti,ab,kw OR Digital Health:ti,ab,kw OR App:ti,ab,kw OR Client-to-provider telemedicine:ti,ab,kw OR Digital biomarkers:ti,ab,kw OR Digital therapeutics:ti,ab,kw OR mobile terminal:ti,ab,kw OR smartphone:ti,ab,kw OR mobile application:ti,ab,kw OR smart application:ti,ab,kw OR wearable:ti,ab,kw OR smartwatch:ti,ab,kw OR Virtual Medicine:ti,ab,kw
#9	#7 OR #8
#10	#3 AND #6 AND #9
CINAHL	
S1	MH Multiple Sclerosis
S2	TI (“Sclerosis” OR “MS” OR “Disseminated Sclerosis”)
S3	S1 OR S2.
S4	MH Fatigue
S5	TI (“Asthenia” OR” Frailty” OR” Muscle Weakness”)
S6	S4 OR S5.
S7	MH telemedicine
S8	TI (“Telehealth” OR “Tele-Referral” OR “Tele-Referrals” OR “Mobile Health” OR “mHealth” OR “eHealth” OR “Telecare” OR “Digital Health” OR “App” OR “Client-to-provider telemedicine” OR “Digital biomarkers” OR “Digital therapeutics” OR “mobile terminal” OR “smartphone” OR “mobile application” OR “smart application” OR “wearable” OR “smartwatch” OR “Virtual Medicine”)
S9	S7 OR S8.
S10	S3AND S6 AND S9.
China National Knowledge Infrastructure (CNKI) (Chinese)	
(主题:“多发性硬化症”or“硬化症) and (主题:“疲劳”or“衰弱”or“虚弱”or“肌无力”) and (主题:“远程医疗”or“远程健康”or“移动健康”or“电子健康”or“远程护理”or“数字健康”or“应用程序”or“数字生物标志物”or“数字疗法”or“移动终端”or“智能手机”or“移动应用程序”or“智能应用程序”or“可穿戴设备”or“智能手表”or“虚拟医学)	
WANFANG DATA (Chinese)	
(主题:“多发性硬化症”or“硬化症) and (主题:“疲劳”or“衰弱”or“虚弱”or“肌无力”) and (主题:“远程医疗”or“远程健康”or“移动健康”or“电子健康”or“远程护理”or“数字健康”or“应用程序”or“数字生物标志物”or“数字疗法”or“移动终端”or“智能手机”or“移动应用程序”or“智能应用程序”or“可穿戴设备”or“智能手表”or“虚拟医学)	
VIP database (Chinese)	
(主题:“多发性硬化症”or“硬化症) and (主题:“疲劳”or“衰弱”or“虚弱”or“肌无力”) and (主题:“远程医疗”or“远程健康”or“移动健康”or“电子健康”or“远程护理”or“数字健康”or“应用程序”or“数字生物标志物”or“数字疗法”or“移动终端”or“智能手机”or“移动应用程序”or“智能应用程序”or“可穿戴设备”or“智能手表”or“虚拟医学)	

### Literature inclusion and exclusion criteria

Inclusion criteria were determined according to the PCC (population, concept, context) principles [22]: (i) participants (P): MS patients; (ii) concept (C): involving the provision of fatigue management based on various telemedicine approaches for MS patients; (iii) context (C): fatigue management. Studies with or without control groups were included to comprehensively explore the range of telemedicine interventions for MS-related fatigue.The type of study was limited to original quantitative, qualitative, and mixed-methods studies. Exclusion criteria: (i) Studies not related to telemedicine; (ii) Research protocols, policy opinions, guidelines, etc.; (iii) Full text not available [18].

### Study selection

After removing duplicates using EndNote X9 software, literature screening was performed by two researchers, strictly following the inclusion and exclusion criteria. The title and abstract were reviewed first, and the full text of studies potentially meeting the inclusion criteria was further examined. Any disagreements were discussed to reach an agreement, or a third party was consulted.

### Data extraction

The contents were extracted as follows: author, year, country, study design, sample size, intervention form, functional characteristics, intervention duration, control group intervention form, and outcomes.

## Results

Following an initial database search yielding 1,395 records, 70 studies were selected after duplicate removal and title/abstract screening. Full-text assessment resulted in the final inclusion of 26 English-language publications from 10 countries: United States [23–28], Germany [29–34], the United Kingdom [35,36], Switzerland [37,38], Iran [39,40], Belgium [41,42], Italy [43,44], Turkey [45,46], the Netherlands [47], and India [48]. Study durations ranged from 2 weeks to 12 months. The included studies comprised randomized controlled trials (n = 12) [23,25,27,28,30–32,35,36,45–47], quasi-experimental studies (n = 9) [24,33,37,39,40,42–44,48], descriptive studies (n = 4) [26,29,34,41], and one cohort study(n = 1) [38]. Control groups were present in 17 studies, with the remaining employing single-arm or descriptive frameworks. All 26 manuscripts were original research published between 2016 and 2024. The main characteristics of the included papers are summarized in Table 2.

**Table 2:** Basic characteristics of the literature for inclusion in this analysis (n = 26).

Author (year)	Country	Study design	Sample size IG/CG	Intervention group				Control group	Outcomes
				Intervention form	Intervention form	Duration		Intervention form	
Turner et al. (2016) [25]	US	RCT	31/33	Teleconference	①②③	6 months		Routine care	ACD
Ehde et al. (2018) [28]	US	RCT	75/88	Teleconference	①②	8 weeks		Routine care	ABD
Kratz et al. (2020) [27]	US	RCT	10/10	Teleconference, Wearable device	①②③	8 weeks		Routine care	ABCDE
Zanotto et al. (2024) [23]	US	RCT	44/39	virtual reality	①②③	6 weeks		Routine training	ABC
Tallner et al. (2016) [31]	Germany	RCT	59/67	Application	①②	6 months		Application	AB
**Author** **(year)**	**Country**	**Study design**	**Sample size** **IG/CG**	**Intervention group**				**Control group**	**Outcomes**
				**Intervention form**	**Functional characteristics**	**Duration**		**Intervention form**	
Pöttgen et al. (2018) [32]	Germany	RCT	139/136	Online platform	①②③	12 weeks			ABD
Flachenecker et al. (2020) [30]	Germany	RCT	34/30	Application Teleconference	①②③	3 months		Routine care	AC
Moss-Morris et al. (2012) [35]	UK	RCT	23/17	Online platform, Teleconference	①②③	10 weeks		Routine care	ABD
Plow et al. (2020) [36]	UK	RCT	57/50/63	Teleconference, Wearable device	①③	12 weeks		Teleconference	AC
Kahraman et al. (2018) [46]	Turkey	RCT	39/39	Application, Teleconference	①	8 weeks		Routine care	ABCD
Eldemir et al. (2023) [45]	Turkey	RCT	15/15	Application, Teleconference	①②	6 weeks		Routine care	AB
De Gier et al. (2023) [47]	Netherlands	RCT	62/64	Teleconference, Online platform	①	12 months		Routine care	AB
Wong et al. (2024) [24]	US	Quasi-experimental study	9/-	Text messaging	①②③	12 weeks		/	ABE
Mokhberdezfuli et al. (2021) [39]	Iran	Quasi-experimental study	120/-	Application	①③	/		/	AE
Roshanghiyas et al. (2024) [40]	Iran	Quasi-experimental study	40/40	Online platform	①②③	6 weeks		Routine training	AB
Petracca et al. (2024) [44]	Italy	Quasi-experimental study	25/26	Teleconference, Game console	①③	6 weeks		Routine care	ABC
Vestito et al. (2024) [43]	Italy	Quasi-experimental study	20/-	Online platform	①③	/		/	AE
**Author** **(year)**	**Country**	**Study design**	**Sample size** **IG/CG**	**Intervention group**				**Control group**	**Outcomes**
				**Intervention form**	**Functional characteristics**		**Duration**	**Intervention form**	
Barrios et al. (2022) [37]	Switzerland	Quasi-experimental study	23/19	Application	①		/	Application	ABE
Kumar et al. (2024) [48]	India	Quasi-experimental study	24/-	Application, Teleconference	①②		6 weeks	/	AC
D’hooghe et al. (2018) [42]	Belgium	Quasi-experimental study	75/-	Application, Wearable device	①②③		12 weeks	/	ABDE
Ibrahim et al. (2022) [33]	Germany	Quasi-experimental study	65/-	Wearable device	①		/	/	AC
Palotai et al. (2021) [26]	US	Descriptive study	64/-	Application, Wearable device	①		2 weeks	/	ABCD
Müller et al. (2021) [34]	Germany	Descriptive study	88/31	Wearable device	①		/	Wearable device	AC
Mäcken et al. (2021) [29]	Germany	Descriptive study	/	Application, Wearable device	①②③		/	/	AB
Van Geel et al. (2020) [41]	Belgium	Descriptive study	19/-	Application	①③		10 weeks	/	ABC
Moebus et al. (2024) [38]	Switzerland	Cohort study	51/23	Wearable device	①		2 weeks	Wearable device	A

Abbreviations: US:the United States; UK: United Kingdom; RCT: randomized controlled trial; IG: Intervention group; CG: Control group; ①: Remote monitoring; ②: Remote guidance; ③: Remote rehabilitation; A: Fatigue characteristics and effects; B: Health related quality of life; C: Physical activity; D: Mental health; E: Feasibility.

### Intervention forms

The intervention forms of telemedicine include applications [26,29–31,37,39,41,42,45,46,48], wearable devices [26,27,29,33,34,36,38,42], teleconferences [21,23,24,26,31,32,40–44], online platforms [32,35,40,43,47], text messaging [24], virtual reality (VR) [23], and game consoles [44]. A total of seven studies used a single intervention and 19 used two or more approaches, specific descriptions are provided in Table 3. The wearable device, with its integrated accelerometer, is able to accurately collect physiological and activity-related data and transmit it via Bluetooth to a specially designed app for in-depth analysis [33,34,38]. Smartphone-based apps integrate a number of modular scales, combining behavioral change theory, rehabilitation medicine theory, and patient-centered design concepts to provide data monitoring and analysis, rehabilitation training assistance, feedback and interaction, and patient information management [29,37,42]. Most of the applications are real-time, highly interactive, and can be used offline [41]. The VR system provides immersive navigation training for MS patients by projecting virtual environments on a television screen, and enhances training by providing motor and cognitively challenging tasks [23]. Gaming consoles enhance the interactive experience between patients and healthcare professionals through high-resolution images and sound effects [44]. In addition, online platforms, teleconferences, and text messaging also provide rich resources and convenient conditions for distance education guidance and real-time feedback interaction.

**Table 3:** Intervention form of telemedicine.

Intervention form	Study	Contents
virtual reality	1.Zanotto	The participants walk on a treadmill while navigating a virtual environment projected on the TV screen through the VR system.
Teleconference, Wearable device	2.Plow	Teleconference:The 12-week interventions consisted of three or six group teleconference sessions and four individually tailored phone calls; Wearable device:Average daily step count was measured at baseline with a waist-worn Autograph trials accelerometer.
Text messaging	3.Wong	Provide fatigue management tips and collect patient feedback via text messages.
Application, Wearable device	4.D’hooghe	Application:Assess the baseline activity level per patient Wearable device:The device was placed horizontally in a belt pouch around the waist to collect actigraphic telemetric data, measuring activity counts from persons with Multiple Sclerosis.
Teleconference, Game console	5.Petracca	Teleconference: One-on-one remote supervised physical therapy sessions via interactive full-body view video conferencing; Game console:Maximizes the effectiveness of video conferencing modes.
Teleconference	6.Turner	Participants’ health goals are first assessed via phone consultation, then monitored using a device connected to a regular phone line with “store-and-forward” technology.
Teleconference, Application	7.Eldemir	Remote Pilates instruction was provided through video conferences via Application.
Teleconference, Wearable device	8.Kratz	Teleconference:Provides remote exercise guidance; Wearable device:Patient-Reported Outcome Diary(PRO-Diary) :Collect physical activity data.
Teleconference	9.Ehde	Teleconference:One one-on-one conference call per week for eight weeks.
Online platform	10.Pöttgen	The online fatigue management program conveys information using a “simulated dialogue” approach.
Online platform	11.Roshanghiyas	Patients in the intervention group received mobile health education on fatigue reduction strategies using a website.
Online platform, Teleconference	12.De Gier	Online Platform: Patients complete learning and assignments related to fatigue management online, and the platform records participation; Teleconference: The therapist evaluates the participant’s treatment progress and provides personalized support through video consultations.
Online platform	13.Vestito	The patient’s movement progress was monitored through game tasks, which in turn indirectly assessed fatigue and gave rehabilitation strategies.
Application, Teleconference	14.Kumar	Teleconference: articipants receive Pilates exercise tutorials via conference calls or YouTube; Application:The participants were invited to upload their performance video after practice sessions.
Application, Teleconference	15.Flachenecker	Application:Participants used the software to document their exercises and to plan their activities and sessions in a physical activity diary; Teleconference:supervise and manage exercises and activities.
Online platform, Teleconference	16.Moss-Morris	Online platform:Fatigue management courses are offered; Teleconference:clarifying goal setting and progress with goals.
Application	17.Tallner	Provide each participant with a one-on-one exercise strategy.
Application	18.Barrios	Used to measure cognitive fatigue over a short period of time;
Wearable device	19.Müller	The sensors were attached to the forefoot of participants’ dominant leg to complete a walking test.
Wearable device	20.Ibrahim	Wearable sensors are worn to monitor gait data during walking tests.
Wearable device	21.Moebus	Fine-grained modeling of perceived fatigue based on passively collected physiological signals using wear-ables.
Application, Wearable device	22.Palotai	The mobile app; Wrist-worn actigraphic MotionLogger watch:assessed physical activity during the daytime and sleep quality at night throughout the entire study; Nox T3 home sleep test (HST) device:assess sleep apnea and periodic limb movements at one night in the patient’s home.
Application	23.Van Geel	Track walking speed, distance, steps and give feedback to participants.
Application, Teleconference	24.Kahraman	Application: Remote meetings can be scheduled. Teleconference: Motor visualization training at home via remote video conferencing.
Application, Wearable device	25.Mäcken	Application:Fatigue is measured with different patient-reported outcome measures and tests. Wearable device:Capture factors that affect life and the environment.
Application	26.Mokhberdezfuli	Medication time reminder, assessing the severity of fatigue, and calculating the score of the Fatigue Severity Scale.

### Functional characteristics

The functional characteristics of telemedicine include remote monitoring, remote guidance, and remote rehabilitation, details are described in Table 4.

**Table 4:** Functional characteristics of telemedicine.

Functional characteristics	Study	Contents
Remote monitoring	1.Plow	Mental and physical function.
	2.Turner	Fatigue severity;depression; balance; pain; physical activity.
	3.Eldemir	Fatigue severity; fatigue impact; physical function; pain;depression; social function; balance; gait; physical activity.
	4.Kratz	Fatigue severity; fatigue impact; depression;pain; sleep disturbances; physical activity;
	5.De Gier	Fatigue severity; pain; emotional health; social function; cognitive function; physical activity;
	6.Zanotto	Fatigue impact; overall quality of life; physical activity.
	7.Ehde	Fatigue effect; pain;depression; gait.
	8.Wong	Fatigue severity.
	9.D’hooghe	Fatigue effect;overall quality of life; physical activity.
	10.Barrios	Cognitive fatigability.
	11.Petracca	Fatigue severity;fatigue effect;overall quality of life.
	12.Roshanghiyas	Fatigue severity;fatigue effect.
	13.Ibrahim	Fatigue severity;gait.
	14.Palotai	Circadian rhythms of fatigue;mood symptoms.
	15.Mäcken	Fatigue severity; heart rate;stress level; sleep disorder
	16.Van Geel	Fatigue severity; fatigue effect;psychological and physiological impacts;overall quality of life;Walking speed; distance;step count.
	17.Müller	Gait parameters
	18.Moebus	Physical; cardiac; and electrodermal activity; skin temperature
	19.Pöttgen	Fatigue severity;
	20.Mokhberdezfuli	Fatigue severity;
	21.Kumar	Fatigue effect;motor function
	22.Flachenecker	Fatigue severity.
	23.Moss-Morris	Fatigue severity;fatigue effect;depression.
	24.Tallner	Fatigue severity;overall quality of life;muscle strength; aerobic capacity;lung Function;physical activity.
	25.Kahraman	Fatigue effect;overall quality of life;depression.
	26.Vestito	Numbness or spasms in the body or limbs.
Remote guidance	1.Turner	Exercise plans;exercise demonstrations; physical activity recommendations.
	2.Eldemir	The basic principles and exercise methods of Pilates.
	3.Kratz	Endurance training, resistance training, and functional exercise demonstrations and guidance.
	4.Zanotto	Exercise training program.
	5.Ehde	Cognitive behavioral strategies;MS fatigue knowledge.
	6.Wong	Fatigue awareness and management techniques, energy-saving methods.
	7.D’hooghe	Standardized recommendations and guidance for energy management.
	8.Roshanghiyas	Fatigue management strategy; energyconservation methods.
	9.Mäcken	MS fatigue knowledge; emotional regulation techniques, and exercise and energy conservation management strategies.
	10.Pöttgen	Fatigue management strategies based on cognitive behavioral therapy.
	11.Moss-Morris	Fatigue management strategies based on cognitive behavioral therapy.
	12.Tallner	Strength training sessions
	13.Flachenecker	Web- and telephone-based, behavior-oriented physical activity coaching.
	14.Kumar	Exercise session videos.
Remote rehabilitation	1.Plow	Teach pedometer walking plan, goal setting and other content, and carry out health education on fatigue management.
	2.Turner	Improve physical activity through telephone counseling sessions and remote health home monitoring based on the principles of motivational interviewing.
	3.Kratz	Provide personalized exercise guidance based on participants’ gait, fatigue level, and other specific conditions.
	4.Zanotto	Provide appropriate exercise and cognitive challenge tasks based on the participant’s performance level.
	5.Wong	Develop personalized recovery text messages based on the needs of the target population.
	6.D’hooghe	Provide standardized energy management advice and guidance based on test results.
	7.Petracca	One on one remote supervised exercise therapy through interactive full-body video conferencing.
	8.Roshanghiyas	Build a special rehabilitation website to upload rehabilitation training content regularly.
	9.Mäcken	Regularly measure fatigue, participate in classes, exercise according to individual candidates, and use energy saving techniques.
	10.Van Geel	Track walking activity through the app, set personalized goals, and provide real-time feedback.
	11.Pöttgen	Based on CBT strategy, the fatigue intervention scheme was delivered through “simulated dialogue” technology.
	12.Mokhberdezfuli	Patients regularly participate in MS related courses and tests, and doctors timely adjust personalized rehabilitation programs according to feedback.
	13.Vestito	Develop accurate game rehabilitation treatment plan for patients.
	14.Flachenecker	Develop personalized exercise programs according to individual goals and health conditions of patients.
	15.Moss-Morris	Online course customization based on cognitive behavioral therapy.

The severity and impact of fatigue, health-related quality of life factors like pain, depression, physical function, cognitive function, and sleep disorders, and physical activity metrics like gait, balance, muscle strength, activity duration, frequency, and intensity are the three main areas of data that are monitored by the 26 studies that reported on remote monitoring [17,20–44]. For example, studies such as Barrios used numerical sign-matching logic to automatically record fatigue test results from MS patients, systematically analyzing the level of fatigue and its correlation with other clinical data [37]. Studies have also continuously optimized assessment tools through patient feedback to ensure the validity of personalized tests [29,31,32].

Remote guidance was reported in 14 research [23–25,27–32,35,40,42,45,48], providing patients with health guidance in both fatigue management and rehabilitation exercises through various forms such as online courses and treatment manuals. Fatigue management covers fatigue knowledge, cognitive behavioral therapy, energy management skills, etc. Rehabilitation exercise guidance includes the types, frequencies and intensities of exercises suitable for MS patients. All the guidance contents are based on systematic literature review and expert opinions to ensure the scientificity and safety of the intervention [29].

The remote rehabilitation was covered in 15 research [23–25,27,29,30,32,35,36,39–44]. At the implementation level, personalized plans are constructed based on the baseline characteristics of patients, integrating core modules such as self-goal setting and task management, promoting dynamic communication between doctors and patients as well as among patients, and achieving plan optimization and strengthened peer support [23–25,27,29,32,35,36,41,42,44]. At the level of psychological intervention, the application of positive expectation orientation and cognitive reconstruction techniques effectively alleviates fatigue-related anxiety and depression emotions [25].

### Outcomes

An analysis of 26 studies that included 43 outcome metrics demonstrated the dual impact of telemedicine on clinical outcomes and implementation feasibility of MS treatments [17,20–44], as detailed in Table 5.

**Table 5:** Outcomes of telemedicine.

Outcome	Study	Tool
Fatigue characteristics and effects	Plow, Eldemir	1.Fatigue Impact Scale (FIS)
	Van Geel, Plow, Flachenecker	2.Multiple Sclerosis Impact Scale (MSIS)
	Zanotto	3.frailtyindex (FI)
	Barrios, D’hooghe, Moebus, Pöttgen	4.Fatigue Scale for Motorand Cognitive Functions (FSMC)
	D’hooghe, Moebus	5.Visual Analogue Scale (VAS)
	Van Geel, Turner, D’hooghe, Wong, Palotai, Ehde, Zanotto, Kumar, Moss-Morris, Kahraman	6.Modified Fatigue Impact Scale (MFIS)
	Wong, De Gier	7.PROMIS Short Form v1.0 Fatigue 8a (Patient-Reported Outcomes Measurement Information System Short Form v1.0 Fatigue 8a)
	Wong	8.PROMIS Short Form v1.0 Self-Efficacy for Managing Symptoms 8a
	Kratz, Van Geel, Moebus, Palotai, Petracca, Eldemir, De Gier, Roshanghiyas, Mokhberdezfuli, Moss-Morris	9.Fatigue Severity Scale (FSS)
	Kratz	10.Fatigue Self Efficacy Scale(FSES)
	Müller	11.aself-report measuread dressing fatigue
	Mäcken, Pöttgen	12.Chalder Fatigue Scale(CFS)
	Ibrahim	13.Borg Rating of Perceived Exertion (RPE)
	De Gier	14.Checklist Individual Strength (CIS)
	Flachenecker, Tallner	15.Würzburger Fatigue Inventory for Multiple Sclerosis(WEIMuS)
Health related quality of life	Ehde	1.Brief Pain Inventory(BPI)
	Kratz	2.The PROMIS Pain Intensity Short Form 3a
	Eldemir, Petracca, Zanotto, Kahraman	3.Multiple Sclerosis Quality of Life-54 (MSQOL-54)
	Van Geel, D’hooghe, De Gier	4.36-Item Short Form Survey(SF-36)
	Kratz, Wong	5.PROMIS Sleep Disturbance Short Form 8a
	Mäcken, Petracca, Kahraman	6.Symbol Digit Modalities Test(SDMT)
	Barrios, D’hooghe, Roshanghiyas, Moss-Morris	7.Expanded Disability Status Scale (EDSS)
	Palotai, Pöttgen	8.Quality of Life in Neurological Disorders(Neuro-QoL)
	Tallner	9.Hamburg Quality of Life Questionnaire for Multiple Sclerosis (HAQUAMS)
Outcome	Study	Tool
Physical activity	Kratz, Turner, Palotai, Plow	1.Godin Leisure-Time Exercise Questionnaire (GLTEQ)
	Van Geel, Zanotto	2.International Physical Activity Questionnaire(IPAQ)
	Ibrahim, Müller, Van Geel, Zanotto, Kahraman	3.Timed 25 FootWalk (T25FW)
	Ibrahim, Müller, Van Geel, Zanotto	4.The six-minute walking test (6-MWT)
	Müller, Petracca	5.Biodex Balance System-BioSway (BBS)
	Van Geel, Kumar, Kahraman	6.Multiple Sclerosis Walking Scale-12(MSWS-12)
	Flachenecker	7.2min/10m walking test
Mental health	D’hooghe,Moss-Morris, Kahraman, Pöttgen	1.Hospital Anxiety Depression Scale (HADS)
	Kratz, Ehde, Turner	2.Patient Health Questionnaire (PHQ-9)
	Palotai	3.The Neuro-QoL anxiety questionnaire
	Palotai	4.The Neuro-QoL depression questionnaire
	Palotai	5.Symptoms of Depression Questionnaire (SDQ)
Feasibility	Vestito	1.prescription adherence
	Wong	2.Patient Activation Measure short form (PAM-13)
	Kratz, Vestito	3.Number of sessions
	D’hooghe	4.D-Quest 2.0
	Barrios	5.response time and calibrated rate
	Mokhberdezfuli	6.Overall reaction to the software
	Kratz, Wong	7.Client Satisfaction Questionnaire (CSQ)

All 26 studies evaluated fatigue management through effective tools such as the fatigue severity scale (FSS), modified fatigue impact scale (MFIS), and fatigue scale for motor and cognitive functions (FSMC). Twenty studies reported significant reductions in fatigue severity, duration, and functional limitations [23–25,27,28,30,32,35–38,40–48]. Of these, five demonstrated high effect sizes [27,35,40,41,46], while the remaining showed low-to-moderate effects [23–25,32,33]. Notably, one study showed no fatigue improvement due to baseline levels below the clinical threshold [31]. Five studies that focused solely on device effects were excluded from the outcome analysis due to insufficient clinical endpoints [26,29,33,34,39].

Meanwhile, Among 17 studies using scales such as the multiple sclerosis quality of life-54 (MSQOL-54) and 36-item short form survey (SF-36) [23,24,26–29,31,32,35,37,40–42,44–47], 12 documented improvements in multidimensional quality of life, including pain relief, cognitive enhancement, and social participation [24,25,27,29,30,32,41,42,44–47]. Assessments of motor function in 12 studies demonstrated improvements in physical ability through standardized walking tests [23,25–27,30,33,34,36,41,44,46,48], although with one notable exception, highlighting significant improvements in limb strength, reported limited improvements in gait speed and endurance [41]. It was also shown that there was no significant difference between the short and long gait tests in fatigue assessment [33,34], so the researchers recommended replacing the long gait test with the short gait test to shorten the assessment time. Mental health assessments across eight studies demonstrated significant findings [25–28,32,35,42,46]. Among these, five studies reported measurable reductions in both anxiety and depression symptoms [28,32,35,42,46],with evidence that physical activity can bring such psychological benefits [25].

In addition, the feasibility of implementing telemedicine was uniformly confirmed in six studies through adherence indicators such as the patient activation measure-13 (PAM-13) and satisfaction indicators including client satisfaction questionnaire (CSQ) scores [24,27,37,39,42,43].

## Discussion

### Initial success and potential of telemedicine in MS fatigue management

Telemedicine overcomes the temporal-spatial limitations of traditional rehabilitation and enables real-time communication and feedback between patients and healthcare professionals [28]. Telemedicine improves patient compliance, self-management, physical functioning, and quality of life. It has shown particular effectiveness in managing fatigue related to multiple sclerosis, specifically reflected in predicting fatigue levels [26,29,38], monitoring fatigue changes [23,27,28,40–42], quantifying the impact of fatigue [25–27,32], improving fatigue management strategies [24,25,36], and enhancing self-efficacy [28].According to research, one of the main ways that remote treatments reduce tiredness may be via modifying neuroplasticity [44]. Specifically, remote exercise and cognitive rehabilitation could induce cortical reorganization, functional rearrangement of neural connections, and changes in the microstructural characteristics of white matter [44]. Notably, MS patients are younger, less impaired, and in a better position to use digital health services than those with many other chronic diseases. Flachenecker pointed out that the positive impact of remote rehabilitation on fatigue can be maintained for 3–6 months through internet-based physical activity [30]. The study confirmed no significant difference between remote intervention and on-site rehabilitation in improving fatigue [27]. This is in line with Wiley’s results, who also discovered that, in comparison to on-site rehabilitation, telemedicine generally offered high-quality therapy [49]. These findings suggest that telemedicine holds potential as a complementary approach to conventional on-site rehabilitation for MS-related fatigue management. However, direct comparative evidence of superiority remains limited, and further positive trials are needed to validate its role as a standalone substitute.

### Further research needed on telemedicine’s effectiveness in MS fatigue and influencing factors

#### Debate over the efficacy of therapy and contributing variables.

Although telemedicine has demonstrated some early success in managing fatigue in MS patients, further research is needed to fully establish its efficacy and identify contributing factors. The effectiveness of telemedicine remains inconclusive: although a trend toward fatigue management exists, only five studies reported high effect sizes [27,35,40,41,46], while the remaining studies reported low to moderate effect sizes. Moreover, these changes did not always translate into significant improvements in clinically relevant outcomes. Following cognitive behavioral treatment, individuals in the intervention group in De Gier’s study received remote online reinforcement [47]. In contrast to the control group, a one-year follow-up revealed no discernible difference in tiredness improvement [47]. Tallner’s study found no improvement in patient fatigue following telemedicine, possibly due to baseline fatigue levels already being well below the threshold [31]. Collectively, these findings highlight the uncertainty and complexity surrounding telemedicine’s effectiveness in treating MS-related fatigue.

Existing research has yielded conflicting findings regarding how baseline factors influence intervention efficacy. The study by Wong, Ehde et al. highlights the importance of psychological functioning and patient activation levels, showing that poorer baseline psychological functioning or inadequate goal-setting may diminish the effectiveness of interventions for MS-related fatigue. Conversely, patients with higher activation levels—defined as the skills, knowledge, and confidence to manage health and make medical decisions—experienced more pronounced reductions in fatigue after tele-intervention [24,28]. By contrast, Petracca and Plow et al. found that baseline characteristics did not significantly moderate tiredness [36,44] Further, Moebus reported that fatigue symptoms were more severe in patients with autonomic nervous system dysfunction, noting that factors like sleep quality and cardiac activity exerted differential effects on fatigue across patient subgroups [38]. The authors also suggested that sleep-related biosignal changes could predict next-day fatigue levels.

#### Reasons for differences in treatment effects and directions for improvement.

Disparities in study design, sample composition, and evaluation instruments may give rise to disagreements regarding efficacy and the variables that affect it. Firstly, most included studies have short durations (6–12 weeks) and limited follow-up periods, which hinder understanding of disease dynamics and the long-term effects of interventions, as well as the ability to capture sustained trends in fatigue. Secondly, few studies have included MS patients with severe disability, advanced age, or low educational attainment, potentially due to the challenges and higher risks of delivering tele-rehabilitation guidance to these subgroups [44,50–52]. Additionally, the commonly used visual analog scale (VAS) fatigue scale is sensitive to recent physical activity and does not effectively differentiate between different dimensions of fatigue, limiting comprehensive and accurate assessment of fatigue levels [38].

To address these gaps and establish long-term efficacy evidence, future research should increase sample sizes even more, diversify sample demographics, and extend follow-up periods. Concurrent efforts should include investigating multimodal treatment protocols and developing more precise fatigue assessment tools by integrating subjective reports with objective physiological markers. Furthermore, the application of artificial intelligence technology is strengthened to analyze patient data through machine learning algorithms to achieve accurate prediction of fatigue risk and intelligent recommendation of intervention programs, so as to improve the effectiveness and sustainability of telemedicine in fatigue management of MS patients.

#### Challenges for telemedicine in MS fatigue management.

The application of telemedicine in MS fatigue management still suffers from the following problems: (i) Digital device accessibility: MS patients may experience neurological impairments—such as mobility limitations, visual deficits, cognitive dysfunction, or psychiatric comorbidities—that hinder their ability to use smart devices effectively [53]. (ii) Patient compliance: Telemedicine may have drawbacks when compared to on-site rehabilitation, including a lack of basic equipment, inconsistent caregiver competency levels, and low patient confidence in teletherapy programs, all of which compromise adherence. (iii) Data quality: Patients may self-diagnose incorrectly, experience psychological discomfort, or even receive non-evidence-based therapeutic recommendations as a result of the abundance of digital data and the challenge of assessing its quality [51]. (iv) Privacy and security: The absence of standardized guidelines for telemedicine services, coupled with the need to maintain robust privacy protections for remote healthcare data, leaves patients vulnerable to security risks due to insufficient institutional safeguards.

#### Recommendations for telemedicine in MS fatigue management.

Given the aforementioned difficulties with telemedicine in managing MS fatigue, this study suggests the following: (i) Future developments should prioritize intelligent assistive technologies to accommodate diverse patient needs. For example, integrating eye-tracking and speech-recognition tools can improve telemedicine accessibility for patients with motor or visual impairments. Additionally, designing user-friendly interfaces with multilingual support and personalized tutorials would lower barriers for older adults or individuals with lower educational levels, ensuring intuitive device operation. (ii) While some studies have integrated gamification to enhance patient motivation and engagement, telehealth interventions must align with clinical objectives in healthcare settings [7,26]. Future programs could incorporate behavioral science theories to design more engaging telehealth models, while strengthening medical resource coordination and professional training to improve intervention quality. (iii) Create a standardized procedure for gathering and analyzing data that includes machine learning algorithms for patient data error correction and real-time validation. Implementing intelligent early-warning systems that flag anomalous data or risks to patients and clinicians would mitigate misdiagnosis and ineffective treatments. (iv) Advanced techniques like blockchain technology may be employed in the future to guarantee patient data traceability and immutability. Simultaneously, access control and multi-level data encryption are put in place to ensure that only the appropriate people are allowed to access sensitive data. Establishing interdisciplinary collaboration platforms is essential to integrate medical, technical, and policy resources, enabling the development of standardized remote service systems, institutional safeguards, and continuous iteration of telemedicine apps and devices [54–56].

## Conclusions

This study systematically reviewed the literature on telemedicine for managing fatigue in MS patients, analyzing intervention types, patient functional characteristics, and outcome metrics. Though its effect size needs to be increased and its mechanism of influence is still up for debate, findings consistently demonstrate that telemedicine can alleviate fatigue symptoms in MS patients. To fully understand the dynamic changes of fatigue and to advance the use of telemedicine in the therapy of MS patients’ fatigue, future research should increase the study size, improve the sample structure, and prolong the study and follow-up time.

## Supporting information

S1 Table Search strategy used for each of the databases. (DOCX)

S1 Checklist Preferred Reporting Items for Systematic reviews and Meta-Analyses extension for Scoping Reviews (PRISMA-ScR) Checklist. (DOCX)

## References

[1] Wiendl H, Gold R, Berger T, Derfuss T, Linker R, Mäurer M, et al. Multiple Sclerosis Therapy Consensus Group (MSTCG): position statement on disease-modifying therapies for multiple sclerosis (white paper). Ther Adv Neurol Disord. 2021;14:17562864211039648. doi: 10.1177/17562864211039648 34422112 PMC8377320

[2] Jakimovski D, Bittner S, Zivadinov R, Morrow SA, Benedict RH, Zipp F, et al. Multiple sclerosis. Lancet. 2024;403(10422):183–202. doi: 10.1016/S0140-6736(23)01473-3 37949093

[3] Thompson AJ, Baranzini SE, Geurts J, Hemmer B, Ciccarelli O. Multiple sclerosis. Lancet. 2018;391(10130):1622–36. doi: 10.1016/S0140-6736(18)30481-1 29576504

[4] Shriwash N, Aiman A, Singh P, Basir SF, Shamsi A, Shahid M, et al. Understanding the role of potential biomarkers in attenuating multiple sclerosis progression via multiomics and network-based approach. PLoS One. 2024;19(12):e0314428. doi: 10.1371/journal.pone.0314428 39700118 PMC11658499

[5] Manjaly Z-M, Harrison NA, Critchley HD, Do CT, Stefanics G, Wenderoth N, et al. Pathophysiological and cognitive mechanisms of fatigue in multiple sclerosis. J Neurol Neurosurg Psychiatry. 2019;90(6):642–51. doi: 10.1136/jnnp-2018-320050 30683707 PMC6581095

[6] Knowles LM, Yang B, Mata-Greve F, Turner AP. Perspectives on fatigue management among veterans living with multiple sclerosis. Multiple Sclerosis Related Disorders. 2024;88:105716. doi: 10.1016/j.msard.2024.105716 38880030 PMC11617002

[7] van der Walt A, Butzkueven H, Shin RK, Midaglia L, Capezzuto L, Lindemann M, et al. Developing a digital solution for remote assessment in multiple sclerosis: from concept to software as a medical device. Brain Sci. 2021;11(9):1247. doi: 10.3390/brainsci11091247 34573267 PMC8471038

[8] Abbadessa G, Ponzano M, Bile F, Miele G, Signori A, Cepparulo S, et al. Health related quality of life in the domain of physical activity predicts confirmed disability progression in people with relapsing remitting multiple sclerosis. Mult Scler Relat Disord. 2023;75:104731. doi: 10.1016/j.msard.2023.104731 37163840

[9] Istepanian RSH. Mobile health (m-Health) in retrospect: the known unknowns. Int J Environ Res Public Health. 2022;19(7):3747. doi: 10.3390/ijerph19073747 35409431 PMC8998037

[10] Global strategy on digital health 2020-2025. https://www.who.int/publications/i/item/9789240020924 32284477

[11] Gentili A, Failla G, Melnyk A, Puleo V, Tanna GLD, Ricciardi W, et al. The cost-effectiveness of digital health interventions: a systematic review of the literature. Front Public Health. 2022;10:787135. doi: 10.3389/fpubh.2022.787135 36033812 PMC9403754

[12] Aziz Butt S, Naseer M, Ali A, Khalid A, Jamal T, Naz S. Remote mobile health monitoring frameworks and mobile applications: Taxonomy, open challenges, motivation, and recommendations. Eng Applications Artificial Intelligence. 2024;133:108233. doi: 10.1016/j.engappai.2024.108233

[13] Azar R, Chan R, Sarkisian M, Burns RD, Marcin JP, Gotthardt C, et al. Adapting telehealth to address health equity: Perspectives of primary care providers across the United States. J Telemed Telecare. 2024. doi: 10.1177/1357633x241238780 PMC11416562 38515372

[14] Pierce Pucci JU, Soloria HM, Eye PG. Managing pediatric-onset multiple sclerosis in an austere setting: A case report. J Telemed Telecare. 2025;31(6):903–6. doi: 10.1177/1357633X241235701 38425268

[15] Inan OT, Tenaerts P, Prindiville SA, Reynolds HR, Dizon DS, Cooper-Arnold K, et al. Digitizing clinical trials. npj Digit Med. 2020;3(1). doi: 10.1038/s41746-020-0302-y PMC7395804 32821856

[16] Mun M, Park Y, Hwang J, Woo K. Types and effects of telenursing in home health care: a systematic review and meta-analysis. Telemed J E Health. 2024;30(9):2431–44. doi: 10.1089/tmj.2023.0188 37707998

[17] Schlegel D. Combined telemedicine-first and direct primary care as a promising model of healthcare delivery. J Telemed Telecare. 2024;:1357633X241300725. doi: 10.1177/1357633X241300725 39632732

[18] Patel JJ, Hill A, Lee Z-Y, Heyland DK, Stoppe C. Critical appraisal of a systematic review: a concise review. Crit Care Med. 2022;50(9):1371–9. doi: 10.1097/CCM.0000000000005602 35853198

[19] Colquhoun HL, Levac D, O’Brien KK, Straus S, Tricco AC, Perrier L, et al. Scoping reviews: time for clarity in definition, methods, and reporting. J Clin Epidemiol. 2014;67(12):1291–4. doi: 10.1016/j.jclinepi.2014.03.013 25034198

[20] Peters MDJ, Marnie C, Tricco AC, Pollock D, Munn Z, Alexander L, et al. Updated methodological guidance for the conduct of scoping reviews. JBI Evid Synth. 2020;18(10):2119–26. doi: 10.11124/JBIES-20-00167 33038124

[21] Tricco AC, Lillie E, Zarin W, O’Brien KK, Colquhoun H, Levac D, et al. PRISMA Extension for Scoping Reviews (PRISMA-ScR): checklist and explanation. Ann Intern Med. 2018;169(7):467–73. doi: 10.7326/M18-0850 30178033

[22] Lockwood C, Dos Santos KB, Pap R. Practical guidance for knowledge synthesis: scoping review methods. Asian Nurs Res (Korean Soc Nurs Sci). 2019;13(5):287–94. doi: 10.1016/j.anr.2019.11.002 31756513

[23] Zanotto T, Galperin I, Pradeep Kumar D, Mirelman A, Yehezkyahu S, Regev K, et al. Effects of a 6-week treadmill training with and without virtual reality on frailty in people with multiple sclerosis. Archives Physical Med Rehabilitation. 2025;106(2):187–94. doi: 10.1016/j.apmr.2024.09.010 PMC13095348 39341443

[24] Wong AWK, Tomazin R, Walker K, Heeb Desai R, Hollingsworth H, Newland PK, et al. Text messaging intervention for fatigue self-management in people with stroke, spinal cord injury, and multiple sclerosis: a pilot study. Disabil Health J. 2024;17(2):101549. doi: 10.1016/j.dhjo.2023.101549 38001005 PMC12108049

[25] Turner AP, Hartoonian N, Sloan AP, Benich M, Kivlahan DR, Hughes C, et al. Improving fatigue and depression in individuals with multiple sclerosis using telephone-administered physical activity counseling. J Consult Clin Psychol. 2016;84(4):297–309. doi: 10.1037/ccp0000086 26913621

[26] Palotai M, Wallack M, Kujbus G, Dalnoki A, Guttmann C. Usability of a mobile app for real-time assessment of fatigue and related symptoms in patients with multiple sclerosis: observational study. JMIR Mhealth Uhealth. 2021;9(4):e19564. doi: 10.2196/19564 33861208 PMC8087974

[27] Kratz AL, Atalla M, Whibley D, Myles A, Thurston T, Fritz NE. Calling out MS fatigue: feasibility and preliminary effects of a pilot randomized telephone-delivered exercise intervention for multiple sclerosis fatigue. J Neurol Phys Ther. 2020;44(1):23–31. doi: 10.1097/NPT.0000000000000296 31738192

[28] Ehde DM, Arewasikporn A, Alschuler KN, Hughes AJ, Turner AP. Moderators of treatment outcomes after telehealth self-management and education in adults with multiple sclerosis: a secondary analysis of a randomized controlled trial. Arch Phys Med Rehabil. 2018;99(7):1265–72. doi: 10.1016/j.apmr.2017.12.012 29337024

[29] Mäcken J, Wiegand M, Müller M, Krawinkel A, Linnebank M. A mobile app for measuring real time fatigue in patients with multiple sclerosis: introducing the fimo health app. Brain Sci. 2021;11(9):1235. doi: 10.3390/brainsci11091235 34573257 PMC8465979

[30] Flachenecker P, Bures AK, Gawlik A, Weiland A-C, Kuld S, Gusowski K, et al. Efficacy of an internet-based program to promote physical activity and exercise after inpatient rehabilitation in persons with multiple sclerosis: a randomized, single-blind, controlled study. IJERPH. 2020;17(12):4544. doi: 10.3390/ijerph17124544 32599767 PMC7344392

[31] Tallner A, Streber R, Hentschke C, Morgott M, Geidl W, Mäurer M, et al. Internet-supported physical exercise training for persons with multiple sclerosis-a randomised, controlled study. Int J Mol Sci. 2016;17(10):1667. doi: 10.3390/ijms17101667 27706046 PMC5085700

[32] Pöttgen J, Moss-Morris R, Wendebourg J-M, Feddersen L, Lau S, Köpke S, et al. Randomised controlled trial of a self-guided online fatigue intervention in multiple sclerosis. J Neurol Neurosurg Psychiatry. 2018;89(9):970–6. doi: 10.1136/jnnp-2017-317463 29549193

[33] Ibrahim AA, Flachenecker F, Gaßner H, Rothhammer V, Klucken J, Eskofier BM, et al. Short inertial sensor-based gait tests reflect perceived state fatigue in multiple sclerosis. Mult Scler Relat Disord. 2022;58:103519. doi: 10.1016/j.msard.2022.103519 35063910

[34] Müller R, Hamacher D, Hansen S, Oschmann P, Keune PM. Wearable inertial sensors are highly sensitive in the detection of gait disturbances and fatigue at early stages of multiple sclerosis. BMC Neurol. 2021;21(1):337. doi: 10.1186/s12883-021-02361-y 34481481 PMC8418019

[35] Moss-Morris R, McCrone P, Yardley L, van Kessel K, Wills G, Dennison L. A pilot randomised controlled trial of an Internet-based cognitive behavioural therapy self-management programme (MS Invigor8) for multiple sclerosis fatigue. Behav Res Ther. 2012;50(6):415–21. doi: 10.1016/j.brat.2012.03.001 22516321

[36] Plow M, Motl RW, Finlayson M, Bethoux F. Response heterogeneity in a randomized controlled trial of telerehabilitation interventions among adults with multiple sclerosis. J Telemed Telecare. 2022;28(9):642–52. doi: 10.1177/1357633X20964693 33100184

[37] Barrios L, Amon R, Oldrati P, Hilty M, Holz C, Lutterotti A. Cognitive fatigability assessment test (cFAST): Development of a new instrument to assess cognitive fatigability and pilot study on its association to perceived fatigue in multiple sclerosis. Digit Health. 2022;8:20552076221117740. doi: 10.1177/20552076221117740 36046638 PMC9421030

[38] Moebus M, Gashi S, Hilty M, Oldrati P, Holz C. Meaningful digital biomarkers derived from wearable sensors to predict daily fatigue in multiple sclerosis patients and healthy controls. iScience. 2024;27(2):108965. doi: 10.1016/j.isci.2024.108965 38362266 PMC10867654

[39] Mokhberdezfuli M, Ayatollahi H, Naser Moghadasi A. A Smartphone-based application for self-management in multiple sclerosis. J Healthc Eng. 2021;2021:6749951. doi: 10.1155/2021/6749951 34221301 PMC8225446

[40] Roshanghiyas S, Sharifi S, Faghihi H, Jahantigh M. Effect of mobile health self-care training on fatigue in multiple sclerosis patients. Med Surg Nurs J. 2024;12(2). doi: 10.5812/msnj-144605

[41] Van Geel F, Geurts E, Abasıyanık Z, Coninx K, Feys P. Feasibility study of a 10-week community-based program using the WalkWithMe application on physical activity, walking, fatigue and cognition in persons with Multiple Sclerosis. Mult Scler Relat Disord. 2020;42:102067. doi: 10.1016/j.msard.2020.102067 32371377

[42] D’hooghe M, Van Gassen G, Kos D, Bouquiaux O, Cambron M, Decoo D, et al. Improving fatigue in multiple sclerosis by smartphone-supported energy management: The MS TeleCoach feasibility study. Mult Scler Relat Disord. 2018;22:90–6. doi: 10.1016/j.msard.2018.03.020 29649789

[43] Vestito L, Ferraro F, Iaconi G, Genesio G, Bandini F, Mori L, et al. STORMS: a pilot feasibility study for occupational TeleRehabilitation in multiple sclerosis. Sensors. 2024;24(19):6470. doi: 10.3390/s24196470 39409510 PMC11479386

[44] Petracca M, Petsas N, Sellitto G, Ruotolo I, Livi C, Bonanno V, et al. Telerehabilitation and onsite rehabilitation effectively improve quality of life, fatigue, balance, and cognition in people with multiple sclerosis: an interventional study. Front Neurol. 2024;15:1394867. doi: 10.3389/fneur.2024.1394867 39175758 PMC11338795

[45] Eldemir K, Guclu-Gunduz A, Eldemir S, Saygili F, Ozkul C, Irkec C. Effects of Pilates-based telerehabilitation on physical performance and quality of life in patients with multiple sclerosis. Disabil Rehabil. 2024;46(9):1807–14. doi: 10.1080/09638288.2023.2205174 37147864

[46] Kahraman T, Savci S, Ozdogar AT, Gedik Z, Idiman E. Physical, cognitive and psychosocial effects of telerehabilitation-based motor imagery training in people with multiple sclerosis: a randomized controlled pilot trial. J Telemed Telecare. 2019;26(5):251–60. doi: 10.1177/1357633x18822355 30744491

[47] de Gier M, Beckerman H, Twisk JW, Knoop H, de Groot V. Effectiveness of a blended booster programme for the long-term outcome of cognitive behavioural therapy for MS-related fatigue: a randomized controlled trial. Mult Scler. 2023;30(1):71–9. doi: 10.1177/13524585231213258 38018811 PMC10782645

[48] Kumar G, Kaur D, Singh AK. Can tele-physiotherapy help in filling treatment gaps during pandemics in multiple sclerosis? J Family Med Prim Care. 2024;13(10):4510–6. doi: 10.4103/jfmpc.jfmpc_204_24 39629369 PMC11610888

[49] Wiley K, Pugh A, Brown-Podgorski BL, Jackson JR, McSwain D. Associations between telemedicine use barriers, organizational factors, and physician perceptions of care quality. Telemed J E Health. 2024;30(12):2883–9. doi: 10.1089/tmj.2024.0249 39229753 PMC11807871

[50] LeeM, NamS. Telehealth utilization among patients with chronic disease: insights from the 2022 Health Information National Trends Survey. J Telemed Telecare. 2024. doi: 10.1177/1357633x24128915839501649

[51] Heesen C, Berger T, Riemann-Lorenz K, Krause N, Friede T, Pöttgen J, et al. Mobile health interventions in multiple sclerosis: a systematic review. Mult Scler. 2023;29(14):1709–20. doi: 10.1177/13524585231201089 37897326 PMC10687804

[52] Yi M, Hui Y, Hu L, Zhang W, Wang Z. The experiences and perceptions of older adults with multimorbidity toward E-Health Care: a qualitative evidence synthesis. Telemed J E Health. 2024;30(10):2527–44. doi: 10.1089/tmj.2024.0211 38920002

[53] De Angelis M, Lavorgna L, Carotenuto A, Petruzzo M, Lanzillo R, Brescia Morra V, et al. Digital technology in clinical trials for multiple sclerosis: systematic review. J Clin Med. 2021;10(11):2328. doi: 10.3390/jcm10112328 34073464 PMC8199078

[54] Howard Z, Win KT, Guan V. Mobile apps used for people living with multiple sclerosis: a scoping review. Mult Scler Relat Disord. 2023;73:104628. doi: 10.1016/j.msard.2023.104628 37003008

[55] Pinarello C, Elmers J, Inojosa H, Beste C, Ziemssen T. Management of multiple sclerosis fatigue in the digital age: from assessment to treatment. Front Neurosci. 2023;17:1231321. doi: 10.3389/fnins.2023.1231321 37869507 PMC10585158

[56] Chike-Harris KE, Durham C, Logan A, Smith G, DuBose-Morris R. Integration of telehealth education into the health care provider curriculum: a review. Telemed J E Health. 2021;27(2):137–49. doi: 10.1089/tmj.2019.0261 32250196

