# Supplementary material for: Application of telemedicine in fatigue management for patients with multiple sclerosis: A scoping review
Source: PLoS One. 2025 Jul 17;20(7):e0327563. doi: 10.1371/journal.pone.0327563 (PMC12270147; doi:10.1371/journal.pone.0327563)
Supplement: S1 Table — (DOCX) [file pone.0327563.s001.docx]

**[T](https://journals.plos.org/plosone/article?id=10.1371/journal.pone.0322493#pone.0322493.s001)able 1. Search strategy used for each of the databases.**

| **PubMed** | |
| --- | --- |
| #1 | "Multiple Sclerosis"［Mesh］ |
| #2 | "Sclerosis"[Title/Abstract] OR "MS"[Title/Abstract] OR "Disseminated Sclerosis"[Title/Abstract] |
| #3 | #1 OR #2 |
| #4 | "Fatigue"[Mesh] |
| #5 | "Frailty"[Title/Abstract] OR "Asthenia"[Title/Abstract]OR "Muscle Weakness"[Title/Abstract] |
| #6 | #4 OR #5 |
| #7 | "telemedicine"［Mesh］ |
| #8 | "Telehealth"[Title/Abstract] OR "Tele-Referral"[Title/Abstract] OR "Tele-Referrals"[Title/Abstract] OR "Mobile Health"[Title/Abstract] OR "mHealth"[Title/Abstract] OR "eHealth"[Title/Abstract] OR "Telecare"[Title/Abstract] OR "Digital Health" OR "App"[Title/Abstract] OR "Digital biomarkers"[Title/Abstract] OR "Digital therapeutics"[Title/Abstract] OR "mobile terminal"[Title/Abstract] OR "smartphone"[Title/Abstract] OR "mobile application"[Title/Abstract] OR "smart application"[Title/Abstract] OR "wearable"[Title/Abstract] OR "smartwatch"[Title/Abstract] OR "Virtual Medicine"[Title/Abstract] |
| #9 | #7 OR #8 |
| #10 | #3 AND #6 AND #9 |
| **Web of Science** | |
| #1 | TS=("Multiple Sclerosis" OR "Sclerosis" OR "MS" OR "Disseminated Sclerosis" ) |
| #2 | TS=("Fatigue" OR "Asthenia" OR" Frailty" OR" Muscle Weakness") |
| #3 | TS=("telemedicine" OR "Telehealth" OR "Tele-Referral" OR "Tele-Referrals" OR "Mobile Health" OR "mHealth" OR "eHealth" OR "Telecare" OR "Digital Health" OR "App" OR "Digital biomarkers" OR "Digital therapeutics" OR "mobile terminal" OR "smartphone" OR "mobile application" OR "smart application" OR "wearable" OR "smartwatch" OR "Virtual Medicine") |
| #4 | #1 AND #2 AND #3 |
| **Cochrane** | |
| #1 | MeSH descriptor: [Multiple Sclerosis] explode all trees |
| #2 | (Multiple Sclerosis or Sclerosis or MS orDisseminated Sclerosis):ti,ab,kw |
| #3 | #1OR#2 |
| #4 | MeSH descriptor: [Asthenia] explode all trees |
| #5 | (Frailty or Fatigue or Muscle Weakness):ti,ab,kw |
| #6 | #4OR#5 |
| #7 | #3AND#6 |
| #8 | MeSH descriptor: [Telemedicine] explode all trees |
| #9 | (Telehealth or Tele-Referral or Tele-Referrals or Mobile Health or mHealth or eHealth or Telecare or Digital Health or App or Digital biomarkers or Digital therapeutics or mobile terminal or smartphone or mobile application or smart application or wearable or smartwatch or Virtual Medicine):ti,ab,kw |
| #10 | #8OR#9 |
| #11 | #7AND#10 |
| **Embase** | |
| #1 | 'Multiple Sclerosis'/exp |
| #2 | 'Sclerosis':ti,ab,kw OR 'MS':ti,ab,kw OR 'Disseminated Sclerosis':ti,ab,kw |
| #3 | #1 OR #2 |
| #4 | 'Fatigue'/exp |
| #5 | Asthenia:ti,ab,kw OR Frailty:ti,ab,kw OR Muscle Weakness:ti,ab,kw |
| #6 | #4 OR #5 |
| #7 | 'telemedicine'/exp |
| #8 | Telehealth:ti,ab,kw OR Tele-Referral:ti,ab,kw OR Tele-Referrals:ti,ab,kw OR Mobile Health:ti,ab,kw OR mHealth:ti,ab,kw OR eHealth OR Telecare:ti,ab,kw OR Digital Health:ti,ab,kw OR App:ti,ab,kw OR Digital biomarkers:ti,ab,kw OR Digital therapeutics:ti,ab,kw OR mobile terminal:ti,ab,kw OR smartphone:ti,ab,kw OR mobile application:ti,ab,kw OR smart application:ti,ab,kw OR wearable:ti,ab,kw OR smartwatch:ti,ab,kw OR Virtual Medicine:ti,ab,kw |
| #9 | #7 OR #8 |
| #10 | #3 AND #6 AND #9 |
| **CINAHL** | |
| S1 | MH Multiple Sclerosis |
| S2 | TI ( "Sclerosis" OR "MS" OR "Disseminated Sclerosis" ) |
| S3 | S1 OR S2 |
| S4 | MH Fatigue |
| S5 | TI ("Asthenia" OR" Frailty" OR" Muscle Weakness" ) |
| S6 | S4 OR S5 |
| S7 | MH telemedicine |
| S8 | TI ("Telehealth" OR "Tele-Referral" OR "Tele-Referrals" OR "Mobile Health" OR "mHealth" OR "eHealth" OR "Telecare" OR "Digital Health" OR "App" OR "Digital biomarkers" OR "Digital therapeutics" OR "mobile terminal" OR "smartphone" OR "mobile application" OR "smart application" OR "wearable" OR "smartwatch" OR "Virtual Medicine" ) |
| S9 | S7 OR S8 |
| S10 | S3AND S6 AND S9 |
| **China National Knowledge Infrastructure (CNKI) (Chinese)** | |
| （主题:“多发性硬化症”or“硬化症) and （主题:“疲劳”or“衰弱”or“虚弱”or“肌无力”）and （主题:“远程医疗”or“远程健康”or“移动健康”or“电子健康”or“远程护理”or“数字健康”or“应用程序”or“数字生物标志物”or“数字疗法”or“移动终端”or“智能手机”or“移动应用程序”or“智能应用程序”or“可穿戴设备”or“智能手表”or“虚拟医学） | |
| **WANFANG DATA (Chinese)** | |
| （主题:“多发性硬化症”or“硬化症) and （主题:“疲劳”or“衰弱”or“虚弱”or“肌无力”）and （主题:“远程医疗”or“远程健康”or“移动健康”or“电子健康”or“远程护理”or“数字健康”or“应用程序”or“数字生物标志物”or“数字疗法”or“移动终端”or“智能手机”or“移动应用程序”or“智能应用程序”or“可穿戴设备”or“智能手表”or“虚拟医学） | |
| **VIP database (Chinese)** | |
| （主题:“多发性硬化症”or“硬化症) and （主题:“疲劳”or“衰弱”or“虚弱”or“肌无力”）and （主题:“远程医疗”or“远程健康”or“移动健康”or“电子健康”or“远程护理”or“数字健康”or“应用程序”or“数字生物标志物”or“数字疗法”or“移动终端”or“智能手机”or“移动应用程序”or“智能应用程序”or“可穿戴设备”or“智能手表”or“虚拟医学） | |
