# Supplementary material for: Application of telemedicine in fatigue management for patients with multiple sclerosis: A scoping review
Source: PLoS One. 2025 Jul 17;20(7):e0327563. doi: 10.1371/journal.pone.0327563 (PMC12270147; doi:10.1371/journal.pone.0327563)
Supplement: S1 Checklist — (DOCX) [file pone.0327563.s002.docx]

**Preferred Reporting Items for Systematic reviews and Meta-Analyses extension for Scoping Reviews (PRISMA-ScR) Checklist**

| **SECTION** | **ITEM** | **PRISMA-ScR CHECKLIST ITEM** | **REPORTED ON PAGE #** |
| --- | --- | --- | --- |
| **TITLE** | | | |
| Title | 1 | Identify the report as a scoping review. | Application of Telemedicine in Fatigue Management for Patients  with Multiple Sclerosis: A Scoping Review |
| **ABSTRACT** | | | |
| Structured summary | 2 | Provide a structured summary that includes (as applicable): background, objectives, eligibility criteria, sources of evidence, charting methods, results, and conclusions that relate to the review questions and objectives. | **Abstract**  **Background:** Fatigue is a prevalent symptom in people with Multiple Sclerosis , but evidence for the effectiveness of telemedicine in treating this symptom remains incomplete. Despite favorable clinical trial results, its integration into practice and systematic evaluation is limited.  **Objective:** The purpose of this research project is to carefully assess how well telemedicine works for managing fatigue in MS patients.  **Methods:** This scoping review adhered to the Joanna Briggs Institute methodological framework and followed the PRISMA-ScR reporting guidelines. A search covering literature in both English and Chinese up until December 2024 was carried out in the electronic databases of PubMed, Embase, Web of Science, CINAHL, Cochrane Library, Google Scholar, CNKI, Wan Fang, and Wei Pu. Studies that assessed telemedicine-based therapies for patients with multiple sclerosis and documented fatigue-related outcomes were eligible. The collected literature was compiled, examined, and pertinent information was extracted by two independent reviewers.  **Results:** A total of 26 papers were included, all in English. Applications(n=11), wearable devices(n=8), teleconferences(n=11), online platforms(n=5), text messaging(n=1), virtual reality(n=1), and game consoles(n=1) are some of the intervention forms of telemedicine. Remote monitoring(100% of studies), remote guidance(54%), and remote rehabilitation(58%) are some of the functional characteristics of telemedicine. Fatigue characteristics and its impact, health-related quality of life, physical activity, mental health, and the feasibility of remote interventions are among the outcome indicators. While 77% of studies reported statistically significant fatigue reduction, effect sizes varied from small to moderate.  **Conclusion:** Telemedicine demonstrates potential as a viable alternative to conventional rehabilitation for managing MS-related fatigue, particularly through multimodal interventions enabling personalized and real-time management. However, the heterogeneity in influencing factors and treatment effects warrants validation through large-scale trials. Future research should prioritize multimodal strategies, optimizing sample composition, extending follow-up periods, and integrating standardized assessment tools to enhance intervention precision.  **Keywords:** multiple sclerosis; fatigue; telemedicine; remote rehabilitation; scope review. |
| **INTRODUCTION** | | | |
| Rationale | 3 | Describe the rationale for the review in the context of what is already known. Explain why the review questions/objectives lend themselves to a scoping review approach. | Multiple sclerosis (MS), a chronic autoimmune disease of the central nervous system, represents the leading cause of non-traumatic neurological disability in young adults worldwide. Among the range of symptoms of MS, fatigue is one of the most prevalent and disabling. Current strategies (e.g., medications, exercise programs, CBT) show limited efficacy. Despite the increasing adoption of telemedicine for MS-related fatigue, the heterogeneity of interventions and outcomes limits clinical consensus. The scoping review is essential to systematically map the available evidence, elucidate forms of telemedicine interventions and their functional characteristics, determine the efficacy of interventions, and identify barriers to implementation - ultimately guiding the development of standardized, patient-centered frameworks. |
| Objectives | 4 | Provide an explicit statement of the questions and objectives being addressed with reference to their key elements (e.g., population or participants, concepts, and context) or other relevant key elements used to conceptualize the review questions and/or objectives. | ① What are the forms of intervention of telemedicine in fatigue management of MS patients? ② What are the functional characteristics of telemedicine in fatigue management of MS patients? ③ What are the outcome measures of telemedicine in fatigue management of MS patients? ④ What are the intervention effects of telemedicine in fatigue management of MS patients? |
| **METHODS** | | | |
| Protocol and registration | 5 | Indicate whether a review protocol exists; state if and where it can be accessed (e.g., a Web address); and if available, provide registration information, including the registration number. | This review adhered to PRISMA-ScR guidelines. While a formal protocol was not registered, the methodology (including search strategy, inclusion criteria, and data extraction steps) was predefined and reviewed by all co-authors to ensure rigor. Full methodological details are provided in the *Methods* section. Additional documentation is available upon request. |
| Eligibility criteria | 6 | Specify characteristics of the sources of evidence used as eligibility criteria (e.g., years considered, language, and publication status), and provide a rationale. | Inclusion criteria were determined according to the PCC principles: ① Participants (P): MS patients; ② Concept (C): involving the provision of fatigue management based on various telemedicine approaches for MS patients; ③ Context (C): fatigue management. The type of study was limited to original quantitative, qualitative, and mixed-methods studies. Exclusion criteria: (1) Studies not related to telemedicine; (2) Research protocols, policy opinions, guidelines, etc.; (3) Full text not available. Literature published up to December 2024 was searched. Due to the language limitations of the research team, only English and Chinese literature were included. |
| Information sources* | 7 | Describe all information sources in the search (e.g., databases with dates of coverage and contact with authors to identify additional sources), as well as the date the most recent search was executed. | A search was conducted in the electronic databases PubMed, Embase, Web of Science, CINAHL, Cochrane Library, Google Scholar, CNKI, Wan Fang, and Wei Pu, covering literature in both English and Chinese up to December 2024. |
| Search | 8 | Present the full electronic search strategy for at least 1 database, including any limits used, such that it could be repeated. | Take PubMed for example:“Multiple Sclerosis”［Mesh］ OR “Sclerosis”［Ti/Ab］ OR “MS”［Ti/Ab］ OR “Disseminated Sclerosis”）AND（“Asthenia”[Mesh]OR “Frailty”［Ti/Ab］OR “Fatigue”［Ti/Ab］OR “Muscle Weakness”［Ti/Ab］）AND（“telemedicine”［Mesh］OR “telemedicine *”［Ti/Ab］ OR “Telehealth”［Ti/Ab］OR“Tele-Referral”［Ti/Ab］OR“Tele-Referrals”［Ti/Ab］OR“/Mobile Health”［Ti/Ab］OR“mHealth”［Ti/Ab］OR“eHealth”［Ti/Ab］OR“Telecare”［Ti/Ab］OR“Digital Health”OR［Ti/Ab］OR“App”［Ti/Ab］OR“Client-to-provider telemedicine”［Ti/Ab］OR“Digital biomarkers”［Ti/Ab］OR“Digital therapeutics”［Ti/Ab］OR“mobile terminal”［Ti/Ab］OR“smartphone”［Ti/Ab］OR“mobile application”［Ti/Ab］OR“smart application”［Ti/Ab］OR“wearable”［Ti/Ab］OR“smartwatch”［Ti/Ab］OR“Virtual Medicine”［Ti/Ab］ |
| Selection of sources of evidence† | 9 | State the process for selecting sources of evidence (i.e., screening and eligibility) included in the scoping review. | The selection form was developed based on the PCC (Participants, Concept, Context) framework to standardize the inclusion and exclusion criteria.The form also captured study design, intervention type, functional characteristics, and outcome measures.To ensure clarity and consistency, the form was pilot-tested on 20 randomly selected articles from the initial search results.Prior to full screening, a calibration exercise was conducted to enhance inter-reviewer agreement. Two independent reviewers screened the same 50 articles using the revised form. Discrepancies were discussed in a consensus meeting, and the form was further adjusted to address ambiguities.  Two reviewers independently screened titles and abstracts of 1,395 identified records after duplicate removal. Articles meeting the PCC criteria advanced to full-text review. For the 70 full-text articles assessed, disagreements were resolved through discussion or consultation with a third reviewer. |
| Data charting process‡ | 10 | Describe the methods of charting data from the included sources of evidence (e.g., calibrated forms or forms that have been tested by the team before their use, and whether data charting was done independently or in duplicate) and any processes for obtaining and confirming data from investigators. | Xiaoyan Gong and Xiaoyu Xue jointly developed a data chart table and determined to extract the following variables: The authors, publishing countries, study design, sample size, remote intervention forms and functional characteristics, intervention duration, outcome indicators and outcome indicator measurement tools were all involved in the data extraction work. Xiaoyan Gong and Xiaoyu Xue independently drew data charts and discussed the results using Word. |
| Data items | 11 | List and define all variables for which data were sought and any assumptions and simplifications made. | “We abstracted data on article characteristics (e.g., The authors, publishing countries, study design, sample size， intervention duration), remote intervention forms (e.g., Applications, wearable devices, teleconferences, online platforms, text messaging, virtual reality, and game consoles), functional characteristics(e.g. ,Remote monitoring, remote guidance, and remote rehabilitation), and outcome indicators(e.g. , Fatigue characteristics and its impact, health-related quality of life, physical activity, mental health, and the feasibility of remote interventions).” |
| Critical appraisal of individual sources of evidence§ | 12 | If done, provide a rationale for conducting a critical appraisal of included sources of evidence; describe the methods used and how this information was used in any data synthesis (if appropriate). | This scoping review did not conduct a critical appraisal of individual sources of evidence. The rationale for omitting this step aligns with the methodological framework of scoping reviews, which prioritize mapping the breadth and scope of existing literature rather than synthesizing evidence for clinical decision-making or evaluating methodological quality. The Joanna Briggs Institute (JBI) guidelines for scoping reviews explicitly state that critical appraisal is optional and context-dependent, particularly when the review aims to identify knowledge gaps or characterize interventions rather than assess efficacy or risk of bias.  In this study, the focus was on answering descriptive research questions related to telemedicine intervention forms, functional characteristics, outcome measures, and effects in fatigue management for multiple sclerosis. The inclusion criteria (PCC framework) emphasized relevance to telemedicine-based fatigue management rather than methodological rigor. While the PRISMA-ScR checklist guided reporting, it does not mandate quality assessment for scoping reviews. |
| Synthesis of results | 13 | Describe the methods of handling and summarizing the data that were charted. | The purpose of this research project is to carefully assess how well telemedicine works for managing fatigue in MS patients. The main characteristics of the included papers are summarized in Table 1. The Intervention forms are summarized in Table 2.The Functional characteristics are summarized in Table 3. The Outcomes of Telemedicine are summarized in Table 4. |
| **RESULTS** | | | |
| Selection of sources of evidence | 14 | Give numbers of sources of evidence screened, assessed for eligibility, and included in the review, with reasons for exclusions at each stage, ideally using a flow diagram. | 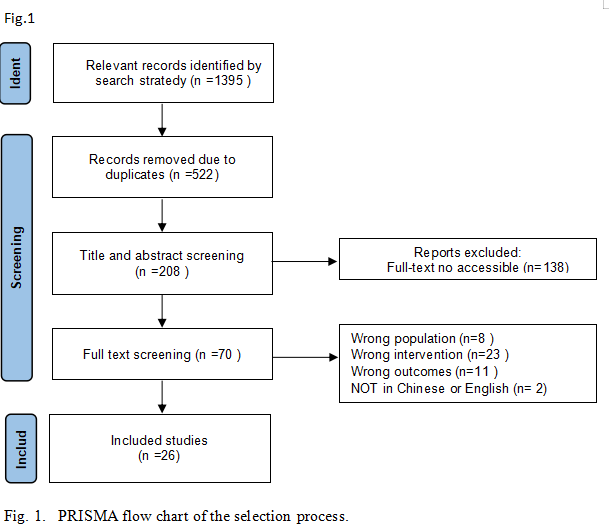 |
| Characteristics of sources of evidence | 15 | For each source of evidence, present characteristics for which data were charted and provide the citations. | The main characteristics(e.g., The authors, publishing countries, study design, sample size， intervention duration) of the included papers are summarized in Table 1.. The Intervention forms(e.g., Applications, wearable devices, teleconferences, online platforms, text messaging, virtual reality, and game consoles) are summarized in Table 2.The Functional characteristics (e.g. ,Remote monitoring, remote guidance, and remote rehabilitation)are summarized in Table 3. The Outcomes of Telemedicine (e.g. , Fatigue characteristics and its impact, health-related quality of life, physical activity, mental health, and the feasibility of remote interventions) are summarized in Table 4. |
| Critical appraisal within sources of evidence | 16 | If done, present data on critical appraisal of included sources of evidence (see item 12). | This scoping review did not conduct a critical appraisal of individual sources of evidence. |
| Results of individual sources of evidence | 17 | For each included source of evidence, present the relevant data that were charted that relate to the review questions and objectives. | 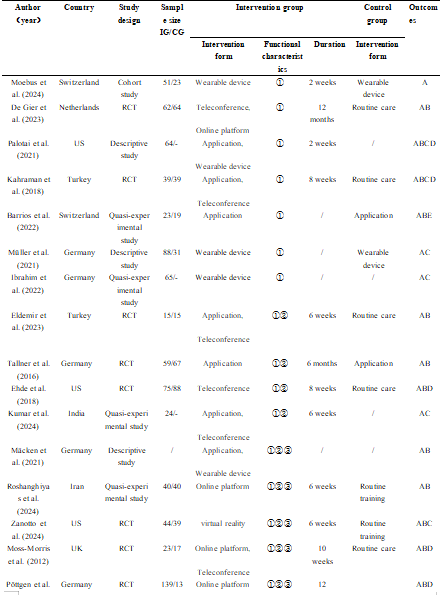  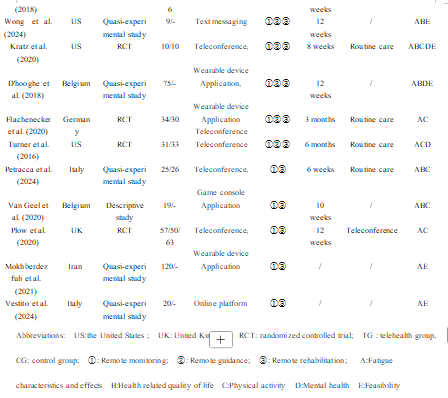 |
| Synthesis of results | 18 | Summarize and/or present the charting results as they relate to the review questions and objectives. | 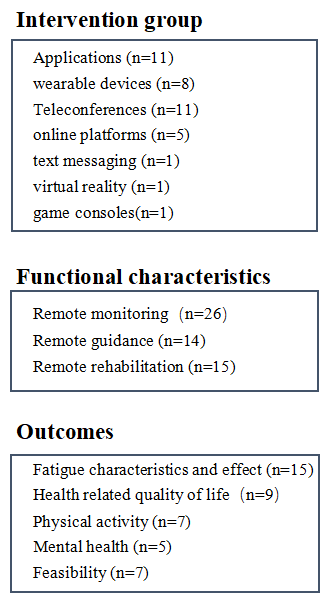 |
| **DISCUSSION** | | | |
| Summary of evidence | 19 | Summarize the main results (including an overview of concepts, themes, and types of evidence available), link to the review questions and objectives, and consider the relevance to key groups. | The present study found that while telerehabilitation demonstrated an overall positive trend in managing fatigue among patients with multiple sclerosis (MS), only a limited number of studies reported high effect sizes, indicating room for efficacy improvement. Additionally, the influencing factors of therapeutic outcomes remain controversial. These discrepancies stem from three key limitations: ① Short study duration (6-12 weeks) hindering long-term efficacy evaluation; ② Sample bias with underrepresentation of older adults, severely disabled individuals, and low-education populations; ③ Suboptimal assessment tools, such as the VAS scale's inability to differentiate multidimensional fatigue features. Future research should implement three strategic improvements: extending follow-up periods (≥24 months), optimizing sample composition to include critical subgroups, and developing comprehensive assessment systems integrating biomarkers with multidimensional fatigue scales. |
| Limitations | 20 | Discuss the limitations of the scoping review process. | The coverage of the literature included in this study is limited: only Chinese and English literature is included, and important evidence in other languages may be missed. |
| Conclusions | 21 | Provide a general interpretation of the results with respect to the review questions and objectives, as well as potential implications and/or next steps. | The telemedicine system based on intelligent technology, through the integration of mobile applications, wearable devices, virtual reality and cloud-based conferencing and other multifaceted tools, has constructed a treatment model that runs through the whole process of “real-time monitoring - dynamic guidance - personalized rehabilitation”, and has shown positive effects in predicting fatigue, monitoring fatigue trends, quantifying the effects of fatigue, and improving fatigue management strategies and self-efficacy. However, the efficacy of telemedicine has much room for improvement, and the factors affecting it are still controversial. In the future, we need to conduct higher-quality studies to extend the follow-up period, optimize the sample structure, and develop an assessment system that combines biomarkers and multidimensional scales. |
| **FUNDING** | | | |
| Funding | 22 | Describe sources of funding for the included sources of evidence, as well as sources of funding for the scoping review. Describe the role of the funders of the scoping review. | The author did not receive any financial support for the research, authorship, and/or publication of this article. The study received no specific grants from any public, commercial, or nonprofit sector. |

JBI = Joanna Briggs Institute; PRISMA-ScR = Preferred Reporting Items for Systematic reviews and Meta-Analyses extension for Scoping Reviews.

* Where *sources of evidence* (see second footnote) are compiled from, such as bibliographic databases, social media platforms, and Web sites.

† A more inclusive/heterogeneous term used to account for the different types of evidence or data sources (e.g., quantitative and/or qualitative research, expert opinion, and policy documents) that may be eligible in a scoping review as opposed to only studies. This is not to be confused with *information sources* (see first footnote).

‡ The frameworks by Arksey and O’Malley (6) and Levac and colleagues (7) and the JBI guidance (4, 5) refer to the process of data extraction in a scoping review as data charting*.*

§ The process of systematically examining research evidence to assess its validity, results, and relevance before using it to inform a decision. This term is used for items 12 and 19 instead of "risk of bias" (which is more applicable to systematic reviews of interventions) to include and acknowledge the various sources of evidence that may be used in a scoping review (e.g., quantitative and/or qualitative research, expert opinion, and policy document).

*From:* Tricco AC, Lillie E, Zarin W, O'Brien KK, Colquhoun H, Levac D, et al. PRISMA Extension for Scoping Reviews (PRISMAScR): Checklist and Explanation. Ann Intern Med. 2018;169:467–473. [doi: 10.7326/M18-0850](http://annals.org/aim/fullarticle/2700389/prisma-extension-scoping-reviews-prisma-scr-checklist-explanation).
