# Supplementary material for: Application of telemedicine in fatigue management for patients with multiple sclerosis: A scoping review
Source: PLoS One. 2025 Jul 17;20(7):e0327563. doi: 10.1371/journal.pone.0327563 (PMC12270147; doi:10.1371/journal.pone.0327563)
Supplement: Attachment — Submitted filename: Response_to_Reviewers_auresp_1.docx [file pone.0327563.s004.docx]

**Dear Editor and Reviewers,**

We sincerely appreciate the time and expertise invested in reviewing our manuscript 【PONE-D-25-18056. Thank you for your constructive feedback, which has significantly strengthened the quality of this work. We have carefully addressed all comments and revised the manuscript accordingly. Below, we provide a point-by-point response to each suggestion, with corresponding revisions highlighted in the tracked-changes version of the manuscript.

**Journal Requirements:**

**1.*“Please ensure that your manuscript meets PLOS ONE's style requirements, including those for file naming. ”***

**Response**: We have revised the manuscript to fully comply with PLOS ONE’s formatting guidelines. Should any formatting inconsistencies persist despite our rigorous checks using the PLOS ONE templates, we remain fully open to implementing additional corrections as directed by the editorial office. A 24-hour revision turnaround is guaranteed upon request.

***2.“Please amend either the title on the online submission form (via Edit Submission) or the title in the manuscript so that they are identical.”***

**Response:** We have ensured consistency between the title in the online submission system and the manuscript. The finalized title is:【Application of telemedicine in fatigue management for patients with multiple sclerosis: A scoping review**】**

***3.“Please include a caption for figure 1.”***

**Response: 【line133】**

A detailed caption for Figure 1 has been added to the manuscript, as shown below:

【Fig 1. PRISMA flow chart of the selection process.】

***4.“Please include captions for your Supporting Information files at the end of your manuscript, and update any in-text citations to match accordingly.”***

**Response:** 【**line 603**】

1. A dedicated paragraph titled "Supporting Information " has been inserted after the References section.
2. Captions for all Supporting Information files (e.g., [S1 Table](S1 Table.docx)) have been added under the "Supporting Information" section at the end of the manuscript.
3. In-text citations to Supporting Information have been standardized

***5.“Please review your reference list to ensure that it is complete and correct. If you have cited papers that have been retracted, please include the rationale for doing so in the manuscript text, or remove these references and replace them with relevant current references. Any changes to the reference list should be mentioned in the rebuttal letter that accompanies your revised manuscript. If you need to cite a retracted article, indicate the article’s retracted status in the References list and also include a citation and full reference for the retraction notice.”***

**Response:** 【line 427-430 line 440-443 Line469-471 Line472-474】

We sincerely appreciate the reviewer's meticulous feedback regarding the integrity of our reference list. In accordance with the comment, we have thoroughly re-examined all cited references and confirm that no retracted articles were included in the manuscript. Furthermore, to strengthen the methodological framework and contextual relevance of our study, we have added four rigorously peer-reviewed publications to the reference list:

【4. Shriwash N, Aiman A, Singh P, Basir SF, Shamsi A, Shahid M, et al. Understanding the role of potential biomarkers in attenuating multiple sclerosis progression via multiomics and network-based approach. PLoS One. 2024;19: e0314428. doi:10.1371/journal.pone.0314428】

【8. Abbadessa G, Ponzano M, Bile F, Miele G, Signori A, Cepparulo S, et al. Health related quality of life in the domain of physical activity predicts confirmed disability progression in people with relapsing remitting multiple sclerosis. Multiple Sclerosis and Related Disorders. 2023;75: 104731. doi:10.1016/j.msard.2023.104731】

【18. Patel JJ, Hill A, Lee Z-Y, Heyland DK, Stoppe C. Critical Appraisal of a Systematic Review: A Concise Review. Crit Care Med. 2022;50: 1371–1379. doi:10.1097/CCM.0000000000005602】

【19. Colquhoun HL, Levac D, O’Brien KK, Straus S, Tricco AC, Perrier L, et al. Scoping reviews: time for clarity in definition, methods, and reporting. J Clin Epidemiol. 2014;67: 1291–1294. doi:10.1016/j.jclinepi.2014.03.013】

This revision ensures our reference list is complete ,correct, and transparent .If the reviewers deem any of these references unnecessary, we will promptly remove them.

**Additional Editor Comments :**

***1.  I suggest further clarification as to why the scoping review format was chosen as opposed to a more in-depth systematic review, considering that “effectiveness” of interventions is also mentioned.***

**Response:**【**line 108-133**】

Considering the exploratory purpose of this study, we fully agree that scope review is the most appropriate method for this study. We added "Type of review" in the "Methods" section of the revised draft, elaborating on the three basic principles of our selection scope review method:

①　Research objective: To explore the multi-dimensional application of telemedicine in MS fatigue management, rather than merely evaluating its effectiveness;

②　Evidence heterogeneity: The high diversity of intervention forms, research designs and outcome indicators;

③　Methodological adaptability: The scope review can draw an evidence map of emerging fields and identify future research directions.

2. ***It is unclear whether studies with or without control groups were included, or both***

**Response:**【**line 152-154 Line173-176**】

Thank you very much for your valuable suggestions. In response to the question you raised regarding the inclusion of studies with or without control groups, we will explain the following three points and ensure they are clearer in the revised version.

① Inclusion criteria: Our scope review includes studies with and without control groups. The PCC framework guided our selection, focusing on the original quantitative, qualitative and mixed approach studies to evaluate telemedicine interventions for fatigue management in MS patients. This method allows for extensive evidence synthesis regardless of the research design.

② Included literature: Among the 26 included studies, 15 were RCTS with clear control groups. 7 were quasi-experimental studies, some with control groups (e.g., Roshanghiyas et al., 2024) and others without (e.g., Mokhberdezfuli et al., 2021). 4 were descriptive/observational studies with control groups (e.g., Muller et al., 2021).

③ Clear expression: We admit the need to improve the transparency of the method. In the revised manuscript, we will clearly state in the methods section that studies with and without control groups are included, because our goal is to map the breadth of the evidence rather than being limited to specific designs. Furthermore, we will further describe the types of studies included in this paper in the results section.

***3. Only the PubMed string is reported, with incomplete syntax and typos. Provide complete strategies (for all databases) in supplementary materials.***

**Response:** 【**line 604**】

We sincerely apologize for the oversight in our initial submission and appreciate the opportunity to improve methodological transparency. We have taken the following corrective actions:

①Complete Search Strategies: As suggested, we have now included the full search strategies for all databases in S1 Table (Table 1 ). Search Strategies, which is provided as Supporting Information.

② Correction of Syntax Errors: We have carefully reviewed and corrected any syntax errors in the PubMed search strategy and standardized the formatting across all databases.

The supplementary file has been prepared and will be submitted alongside the revised manuscript. We hope this addresses your concern and improves reproducibility. Thank you again for your thorough review.

***4. Results are overly descriptive and repetitive in places.***

**Response:**【**line 167-276**】

We sincerely appreciate the reviewer’s valuable feedback regarding the descriptive and repetitive nature of the Results section. We fully agree with the need for greater conciseness and analytical depth, and we have carefully revised the manuscript accordingly. The key modifications include:

① Streamlined Descriptions.

② Enhanced Analysis.

③ Adjust the structure

All changes have been highlighted in the revised manuscript for the reviewer’s convenience. We believe these revisions significantly strengthen the rigor and clarity of the Results section.Should the reviewer identify any remaining areas requiring further refinement, we would be grateful for specific suggestions and are happy to make additional revisions.

***5. The authors state that telemedicine “is anticipated to emerge as a viable substitute for conventional on-site rehabilitation.” (Discussion 317-319). Given the scoping design, these claims need to be tempered or supported by superiority data, which are currently absent.***

**Response:** 【**line 298-302**】

We sincerely appreciate the reviewer’s astute observation regarding the need for cautious interpretation of telemedicine’s role relative to conventional rehabilitation. We agree that the scoping review’s design precludes definitive claims about superiority or substitutability.

**Original statement in Discussion:** "As a result, telemedicine is anticipated to emerge as a viable substitute for conventional on-site rehabilitation."

**Revised version:** "These findings suggest that telemedicine holds potential as a complementary approach to conventional on-site rehabilitation for MS-related fatigue management. However, direct comparative evidence of superiority remains limited, and further positive trials are needed to validate its role as a standalone substitute."

We believe the updated text better reflects the preliminary nature of current evidence while preserving telemedicine’s demonstrated utility. Should the reviewer recommend further tempering of specific claims, we would be grateful for guidance on preferred terminology.
 ***6. Ensure consistency in language editing throughout the manuscript. Occasional grammatical or stylistic adjustments are needed to improve readability. Consider simplifying overly complex sentence structures (e.g., some sections in the results and discussion) to enhance clarity for international audiences. Ensure that all acronyms (e.g., PRO-Diary) are clearly explained in text or table footnotes.***

**Response:** We sincerely appreciate your valuable feedback on enhancing the linguistic quality and readability of our manuscript.We have implemented comprehensive revisions based on your suggestions, with all modifications highlighted in red in the revised manuscript. Key improvements include:

① Systematic Language Refinement：Conducted line-by-line editing to standardize academic expressions. Simplified complex sentence structures in the Results and Discussion sections.

② Structural Clarification：Split lengthy, overly complex sentences to improve clarity.

③ Abbreviation Standardization

**pwMS :** persons with Multiple Sclerosis【**line 209**】

**PRISMA-ScR:** Preferred Reporting Items for Systematic Reviews and Meta-Analyses extension for Scoping Reviews【**line 30-31**】

**PCC :** Population, Concept, Context【**line 149**】

**CNKI:** China National Knowledge Infrastructure【**line 34-35**】

**PRO-Diary:** Patient-Reported Outcome Diary【**line209**】

**PROMIS:** Patient-Reported Outcomes Measurement Information System【**line247**】

**WEIMuS:** Würzburger Fatigue Inventory for Multiple Sclerosis【**line247**】

All acronyms are now explicitly defined upon first use, and consistency has been verified throughout the manuscript. These adjustments align with your feedback and enhance the accessibility of the content for readers. Should further clarification or additional adjustments be required, we are happy to refine the text accordingly.

**Supplementary explanation:**

1. ***"Have the authors made all data underlying the findings in their manuscript fully available?"***

**Response:**【**line 416-417**】

Thank you for emphasizing the importance of data transparency. We confirm full compliance with PLOS ONE’s data policy and have implemented the following revisions:

A "Data Availability" section has been added to the end of the revised manuscript, explicitly stating: "All relevant data are included in the manuscript and its Supporting Information files. The complete search strategies for all databases are provided in S1 File."

2. ***"Upload figures to PACE for format validation."***

**Response:**

We confirm full compliance with the figure formatting requirements through the following actions:

①：PACE Validation Completed：Fig 1 (filename: Fig1.tif) has been successfully processed via the Preflight Analysis and Conversion Engine (PACE).

②：Revised Submission Protocol：The PACE-validated Fig1.tif will replace the original figure file in the resubmitted manuscript.

We are grateful for the opportunity to improve our study through this revision. All changes have been thoroughly cross-checked for compliance with PLOS ONE’s guidelines, including data availability, figure formatting (validated via PACE), and reference integrity. Should additional clarifications or refinements be required, we remain fully committed to addressing them promptly.

Thank you once again for your invaluable input.

Sincerely,

Jie Zheng

Nursing College, Shanxi Medical University

*zhengjie@sxmu.edu.cn*

Tel: +86 18247758920
