# Supplementary material for: Application of telemedicine in fatigue management for patients with multiple sclerosis: A scoping review
Source: PLoS One. 2025 Jul 17;20(7):e0327563. doi: 10.1371/journal.pone.0327563 (PMC12270147; doi:10.1371/journal.pone.0327563)
Supplement: Peer review history

# Peer review: Decision Letter 0

**DOI:** 10.1371/journal.pone.0327563.r001

**Authors:** Gianmarco Abbadessa

Dear Dr. Zheng,

Thank you for submitting your manuscript to PLOS ONE. After careful consideration, we feel that it has merit but does not fully meet PLOS ONE’s publication criteria as it currently stands. Therefore, we invite you to submit a revised version of the manuscript that addresses the points raised during the review process.

Please submit your revised manuscript by Jun 27 2025 11:59PM. If you will need more time than this to complete your revisions, please reply to this message or contact the journal office at plosone@plos.org . When you're ready to submit your revision, log on to https://www.editorialmanager.com/pone/ and select the 'Submissions Needing Revision' folder to locate your manuscript file.

- A rebuttal letter that responds to each point raised by the academic editor and reviewer(s). You should upload this letter as a separate file labeled 'Response to Reviewers'.
- A marked-up copy of your manuscript that highlights changes made to the original version. You should upload this as a separate file labeled 'Revised Manuscript with Track Changes'.
- An unmarked version of your revised paper without tracked changes. You should upload this as a separate file labeled 'Manuscript'.

If applicable, we recommend that you deposit your laboratory protocols in protocols.io to enhance the reproducibility of your results. Protocols.io assigns your protocol its own identifier (DOI) so that it can be cited independently in the future. For instructions see: https://journals.plos.org/plosone/s/submission-guidelines#loc-laboratory-protocols . Additionally, PLOS ONE offers an option for publishing peer-reviewed Lab Protocol articles, which describe protocols hosted on protocols.io. Read more information on sharing protocols at https://plos.org/protocols?utm_medium=editorial-email&utm_source=authorletters&utm_campaign=protocols .

We look forward to receiving your revised manuscript.

Kind regards,

Gianmarco Abbadessa, MD, PhD

Academic Editor

PLOS ONE

Journal Requirements:

When submitting your revision, we need you to address these additional requirements.

1. Please ensure that your manuscript meets PLOS ONE's style requirements, including those for file naming. The PLOS ONE style templates can be found at https://journals.plos.org/plosone/s/file?id=wjVg/PLOSOne_formatting_sample_main_body.pdf and https://journals.plos.org/plosone/s/file?id=ba62/PLOSOne_formatting_sample_title_authors_affiliations.pdf

2. Please amend either the title on the online submission form (via Edit Submission) or the title in the manuscript so that they are identical.

3. Please include a caption for figure 1.

4. Please include captions for your Supporting Information files at the end of your manuscript, and update any in-text citations to match accordingly. Please see our Supporting Information guidelines for more information: http://journals.plos.org/plosone/s/supporting-information.

5. Please review your reference list to ensure that it is complete and correct. If you have cited papers that have been retracted, please include the rationale for doing so in the manuscript text, or remove these references and replace them with relevant current references. Any changes to the reference list should be mentioned in the rebuttal letter that accompanies your revised manuscript. If you need to cite a retracted article, indicate the article’s retracted status in the References list and also include a citation and full reference for the retraction notice.

Additional Editor Comments :

Thank you for submitting your manuscript, “Application of Telemedicine in Fatigue Management for Patients with Multiple Sclerosis: A Scoping Review” (PONE-D-25-18056), to PLOS ONE. We have now received and evaluated the reviewer’s report, and I am pleased to inform you that the manuscript is fundamentally sound and of clear clinical interest. However, a few areas require clarification or minor improvement before we can move forward to publication.

Decision: Minor Revision

Please submit a revised manuscript that addresses each point in a point-by-point Response to Reviewers. Your revision will be assessed to confirm that these minor issues have been resolved.

We look forward to receiving your revised submission.

[Note: HTML markup is below. Please do not edit.]

Reviewers' comments:

Reviewer's Responses to Questions

**Comments to the Author**

1. Is the manuscript technically sound, and do the data support the conclusions?

Reviewer #1: Partly

**********

2. Has the statistical analysis been performed appropriately and rigorously?

Reviewer #1: Yes

**********

3. Have the authors made all data underlying the findings in their manuscript fully available?

The PLOS Data policy

Reviewer #1: No

**********

4. Is the manuscript presented in an intelligible fashion and written in standard English?

Reviewer #1: Yes

**********

Reviewer #1: The article covers a current topic of clinical relevance, providing a comprehensive overview of telemedicine modalities for fatigue management in MS. However, some areas could benefit from improvements in methodological clarity and presentation of results.

- I suggest further clarification as to why the scoping review format was chosen as opposed to a more in-depth systematic review, considering that “effectiveness” of interventions is also mentioned.

- It is unclear whether studies with or without control groups were included, or both

- Only the PubMed string is reported, with incomplete syntax and typos. Provide complete strategies (for all databases) in supplementary materials.

- Results are overly descriptive and repetitive in places.

- The authors state that telemedicine “is anticipated to emerge as a viable substitute for conventional on-site rehabilitation.” (Discussion 317-319). Given the scoping design, these claims need to be tempered or supported by superiority data, which are currently absent.

- Ensure consistency in language editing throughout the manuscript. Occasional grammatical or stylistic adjustments are needed to improve readability. Consider simplifying overly complex sentence structures (e.g., some sections in the results and discussion) to enhance clarity for international audiences. Ensure that all acronyms (e.g., PRO-Diary) are clearly explained in text or table footnotes.

**********

what does this mean? ). If published, this will include your full peer review and any attached files.

If you choose “no”, your identity will remain anonymous but your review may still be made public.

**Do you want your identity to be public for this peer review?** For information about this choice, including consent withdrawal, please see our Privacy Policy

Reviewer #1: No

**********

[NOTE: If reviewer comments were submitted as an attachment file, they will be attached to this email and accessible via the submission site. Please log into your account, locate the manuscript record, and check for the action link "View Attachments". If this link does not appear, there are no attachment files.]

While revising your submission, please upload your figure files to the Preflight Analysis and Conversion Engine (PACE) digital diagnostic tool, https://pacev2.apexcovantage.com/ . PACE helps ensure that figures meet PLOS requirements. To use PACE, you must first register as a user. Registration is free. Then, login and navigate to the UPLOAD tab, where you will find detailed instructions on how to use the tool. If you encounter any issues or have any questions when using PACE, please email PLOS at figures@plos.org

# Author comment: Author response to Decision Letter 1

**DOI:** 10.1371/journal.pone.0327563.r002

23 May 2025

Dear Editor and Reviewers,

We sincerely appreciate the time and expertise invested in reviewing our manuscript 【PONE-D-25-18056. Thank you for your constructive feedback, which has significantly strengthened the quality of this work. We have carefully addressed all comments and revised the manuscript accordingly. Below, we provide a point-by-point response to each suggestion, with corresponding revisions highlighted in the tracked-changes version of the manuscript.

Journal Requirements:

1.“Please ensure that your manuscript meets PLOS ONE's style requirements, including those for file naming. ”

Response: We have revised the manuscript to fully comply with PLOS ONE’s formatting guidelines. Should any formatting inconsistencies persist despite our rigorous checks using the PLOS ONE templates, we remain fully open to implementing additional corrections as directed by the editorial office. A 24-hour revision turnaround is guaranteed upon request.

2.“Please amend either the title on the online submission form (via Edit Submission) or the title in the manuscript so that they are identical.”

Response: We have ensured consistency between the title in the online submission system and the manuscript. The finalized title is:【Application of telemedicine in fatigue management for patients with multiple sclerosis: A scoping review】

3.“Please include a caption for figure 1.”

Response: 【line133】

A detailed caption for Figure 1 has been added to the manuscript, as shown below:

【Fig 1. PRISMA flow chart of the selection process.】

4.“Please include captions for your Supporting Information files at the end of your manuscript, and update any in-text citations to match accordingly.”

Response: 【line 603】

①　A dedicated paragraph titled "Supporting Information " has been inserted after the References section.

②　Captions for all Supporting Information files (e.g., S1 Table) have been added under the "Supporting Information" section at the end of the manuscript.

③　In-text citations to Supporting Information have been standardized

5.“Please review your reference list to ensure that it is complete and correct. If you have cited papers that have been retracted, please include the rationale for doing so in the manuscript text, or remove these references and replace them with relevant current references. Any changes to the reference list should be mentioned in the rebuttal letter that accompanies your revised manuscript. If you need to cite a retracted article, indicate the article’s retracted status in the References list and also include a citation and full reference for the retraction notice.”

Response: 【line 427-430 line 440-443 Line469-471 Line472-474】

We sincerely appreciate the reviewer's meticulous feedback regarding the integrity of our reference list. In accordance with the comment, we have thoroughly re-examined all cited references and confirm that no retracted articles were included in the manuscript. Furthermore, to strengthen the methodological framework and contextual relevance of our study, we have added four rigorously peer-reviewed publications to the reference list:

【4. Shriwash N, Aiman A, Singh P, Basir SF, Shamsi A, Shahid M, et al. Understanding the role of potential biomarkers in attenuating multiple sclerosis progression via multiomics and network-based approach. PLoS One. 2024;19: e0314428. doi:10.1371/journal.pone.0314428】

【8. Abbadessa G, Ponzano M, Bile F, Miele G, Signori A, Cepparulo S, et al. Health related quality of life in the domain of physical activity predicts confirmed disability progression in people with relapsing remitting multiple sclerosis. Multiple Sclerosis and Related Disorders. 2023;75: 104731. doi:10.1016/j.msard.2023.104731】

【18. Patel JJ, Hill A, Lee Z-Y, Heyland DK, Stoppe C. Critical Appraisal of a Systematic Review: A Concise Review. Crit Care Med. 2022;50: 1371–1379. doi:10.1097/CCM.0000000000005602】

【19. Colquhoun HL, Levac D, O’Brien KK, Straus S, Tricco AC, Perrier L, et al. Scoping reviews: time for clarity in definition, methods, and reporting. J Clin Epidemiol. 2014;67: 1291–1294. doi:10.1016/j.jclinepi.2014.03.013】

This revision ensures our reference list is complete ,correct, and transparent .If the reviewers deem any of these references unnecessary, we will promptly remove them.

Additional Editor Comments :

1.  I suggest further clarification as to why the scoping review format was chosen as opposed to a more in-depth systematic review, considering that “effectiveness” of interventions is also mentioned.

Response:【line 108-133】

Considering the exploratory purpose of this study, we fully agree that scope review is the most appropriate method for this study. We added "Type of review" in the "Methods" section of the revised draft, elaborating on the three basic principles of our selection scope review method:

①　Research objective: To explore the multi-dimensional application of telemedicine in MS fatigue management, rather than merely evaluating its effectiveness;

②　Evidence heterogeneity: The high diversity of intervention forms, research designs and outcome indicators;

③　Methodological adaptability: The scope review can draw an evidence map of emerging fields and identify future research directions.

2.It is unclear whether studies with or without control groups were included, or both

Response:【line 152-154 Line173-176】

Thank you very much for your valuable suggestions. In response to the question you raised regarding the inclusion of studies with or without control groups, we will explain the following three points and ensure they are clearer in the revised version.

① Inclusion criteria: Our scope review includes studies with and without control groups. The PCC framework guided our selection, focusing on the original quantitative, qualitative and mixed approach studies to evaluate telemedicine interventions for fatigue management in MS patients. This method allows for extensive evidence synthesis regardless of the research design.

② Included literature: Among the 26 included studies, 15 were RCTS with clear control groups. 7 were quasi-experimental studies, some with control groups (e.g., Roshanghiyas et al., 2024) and others without (e.g., Mokhberdezfuli et al., 2021). 4 were descriptive/observational studies with control groups (e.g., Muller et al., 2021).

③ Clear expression: We admit the need to improve the transparency of the method. In the revised manuscript, we will clearly state in the methods section that studies with and without control groups are included, because our goal is to map the breadth of the evidence rather than being limited to specific designs. Furthermore, we will further describe the types of studies included in this paper in the results section.

3. Only the PubMed string is reported, with incomplete syntax and typos. Provide complete strategies (for all databases) in supplementary materials.

Response: 【line 604】

We sincerely apologize for the oversight in our initial submission and appreciate the opportunity to improve methodological transparency. We have taken the following corrective actions:�

①Complete Search Strategies: As suggested, we have now included the full search strategies for all databases in S1 Table (Table 1 ). Search Strategies, which is provided as Supporting Information.

② Correction of Syntax Errors: We have carefully reviewed and corrected any syntax errors in the PubMed search strategy and standardized the formatting across all databases.

The supplementary file has been prepared and will be submitted alongside the revised manuscript. We hope this addresses your concern and improves reproducibility. Thank you again for your thorough review.

4. Results are overly descriptive and repetitive in places.

Response:【line 167-276】

We sincerely appreciate the reviewer’s valuable feedback regarding the descriptive and repetitive nature of the Results section. We fully agree with the need for greater conciseness and analytical depth, and we have carefully revised the manuscript accordingly. The key modifications include:

① Streamlined Descriptions.

② Enhanced Analysis.

③ Adjust the structure

All changes have been highlighted in the revised manuscript for the reviewer’s convenience. We believe these revisions significantly strengthen the rigor and clarity of the Results section.Should the reviewer identify any remaining areas requiring further refinement, we would be grateful for specific suggestions and are happy to make additional revisions.

5. The authors state that telemedicine “is anticipated to emerge as a viable substitute for conventional on-site rehabilitation.” (Discussion 317-319). Given the scoping design, these claims need to be tempered or supported by superiority data, which are currently absent.

Response: 【line 298-302】

We sincerely appreciate the reviewer’s astute observation regarding the need for cautious interpretation of telemedicine’s role relative to conventional rehabilitation. We agree that the scoping review’s design precludes definitive claims about superiority or substitutability.

Original statement in Discussion: "As a result, telemedicine is anticipated to emerge as a viable substitute for conventional on-site rehabilitation."

Revised version: "These findings suggest that telemedicine holds potential as a complementary approach to conventional on-site rehabilitation for MS-related fatigue management. However, direct comparative evidence of superiority remains limited, and further positive trials are needed to validate its role as a standalone substitute."

We believe the updated text better reflects the preliminary nature of current evidence while preserving telemedicine’s demonstrated utility. Should the reviewer recommend further tempering of specific claims, we would be grateful for guidance on preferred terminology.

6. Ensure consistency in language editing throughout the manuscript. Occasional grammatical or stylistic adjustments are needed to improve readability. Consider simplifying overly complex sentence structures (e.g., some sections in the results and discussion) to enhance clarity for international audiences. Ensure that all acronyms (e.g., PRO-Diary) are clearly explained in text or table footnotes.

Response: We sincerely appreciate your valuable feedback on enhancing the linguistic quality and readability of our manuscript.We have implemented comprehensive revisions based on your suggestions, with all modifications highlighted in red in the revised manuscript. Key improvements include:

① Systematic Language Refinement�Conducted line-by-line editing to standardize academic expressions. Simplified complex sentence structures in the Results and Discussion sections.

② Structural Clarification�Split lengthy, overly complex sentences to improve clarity.

③ Abbreviation Standardization

pwMS : persons with Multiple Sclerosis【line 209】

PRISMA-ScR: Preferred Reporting Items for Systematic Reviews and Meta-Analyses extension for Scoping Reviews【line 30-31】

PCC : Population, Concept, Context【line 149】

CNKI: China National Knowledge Infrastructure【line 34-35】

PRO-Diary: Patient-Reported Outcome Diary【line209】

PROMIS: Patient-Reported Outcomes Measurement Information System【line247】

WEIMuS: Würzburger Fatigue Inventory for Multiple Sclerosis【line247】

All acronyms are now explicitly defined upon first use, and consistency has been verified throughout the manuscript. These adjustments align with your feedback and enhance the accessibility of the content for readers. Should further clarification or additional adjustments be required, we are happy to refine the text accordingly.

Supplementary explanation:

1."Have the authors made all data underlying the findings in their manuscript fully available?"

Response:【line 416-417】

Thank you for emphasizing the importance of data transparency. We confirm full compliance with PLOS ONE’s data policy and have implemented the following revisions:

A "Data Availability" section has been added to the end of the revised manuscript, explicitly stating: "All relevant data are included in the manuscript and its Supporting Information files. The complete search strategies for all databases are provided in S1 File."

2."Upload figures to PACE for format validation."

Response:

We confirm full compliance with the figure formatting requirements through the following actions:

①�PACE Validation Completed�Fig 1 (filename: Fig1.tif) has been successfully processed via the Preflight Analysis and Conversion Engine (PACE).

②�Revised Submission Protocol�The PACE-validated Fig1.tif will replace the original figure file in the resubmitted manuscript.

We are grateful for the opportunity to improve our study through this revision. All changes have been thoroughly cross-checked for compliance with PLOS ONE’s guidelines, including data availability, figure formatting (validated via PACE), and reference integrity. Should additional clarifications or refinements be required, we remain fully committed to addressing them promptly.

Thank you once again for your invaluable input.

Sincerely,

Jie Zheng

Nursing College, Shanxi Medical University

zhengjie@sxmu.edu.cn

Tel: +86 18247758920

Attachment Submitted filename: Response_to_Reviewers_auresp_1.docx

# Editor report: Decision Letter 1

**DOI:** 10.1371/journal.pone.0327563.r003

**Authors:** Gianmarco Abbadessa

Application of telemedicine in fatigue management for patients with multiple sclerosis: A scoping review

PONE-D-25-18056R1

Dear Dr. Zheng,

We’re pleased to inform you that your manuscript, “Application of telemedicine in fatigue management for patients with multiple sclerosis: A scoping review” (PONE-D-25-18056R1), has been judged scientifically suitable for publication and will be formally accepted for publication once it meets all outstanding technical requirements.

Within one week, you’ll receive an e-mail detailing the required amendments. When these have been addressed, you’ll receive a formal acceptance letter and your manuscript will be scheduled for publication.

An invoice will be generated when your article is formally accepted. Please note, if your institution has a publishing partnership with PLOS and your article meets the relevant criteria, all or part of your publication costs will be covered. Please make sure your user information is up-to-date by logging into Editorial Manager® and clicking the **Update My Information**  link at the top of the page. If you have any questions relating to publication charges, please contact our Author Billing department directly at authorbilling@plos.org.

If your institution has a press office, please notify them about your upcoming paper to help maximize its impact. If they’ll be preparing press materials, please inform our press team as soon as possible—no later than 48 hours after receiving the formal acceptance. Your manuscript will remain under strict press embargo until 2 pm Eastern Time on the date of publication. For more information, please contact onepress@plos.org.

Kind regards,

Gianmarco Abbadessa, MD

Academic Editor

PLOS ONE

**********

**Additional Editor Comments**

Thank you for your careful and timely revision of “Application of telemedicine in fatigue management for patients with multiple sclerosis: A scoping review” (PONE-D-25-18056R1). Your point-by-point responses and tracked-changes manuscript show you have fully addressed all of the editors’ and reviewers’ concerns:

- **Manuscript Formatting & Style**
  File naming and figure captions now comply with journal requirements.
- Supporting Information legends have been added and in-text citations standardized.
- Reference list checked and updated; no retracted articles are present.
- **Methodological Transparency**
  Rationale for choosing a scoping review is clearly articulated (research objective, heterogeneity of evidence, methodological adaptability).
- Inclusion criteria for studies with and without control groups are explicitly stated.
- Full search strategies for all databases are provided in S1 Table.
- **Results & Discussion**
  Redundancies removed; narrative is concise and focused.
- Claims about telemedicine’s potential have been appropriately tempered to reflect the scoping design.
- Analytical depth has been enhanced.
- **Language & Readability**
  Grammar, style, and sentence structure have been refined for clarity.
- All acronyms are now defined at first mention.
- **Data Availability & Compliance**
  A “Data Availability” statement has been added.
- Figure 1 has been validated via PACE and replaced with the approved .tif file.

On the basis of these comprehensive revisions, I am pleased to inform you that your manuscript has been accepted for publication in PLOS ONE.

# Editor report: Acceptance letter

**DOI:** 10.1371/journal.pone.0327563.r004

**Authors:** Gianmarco Abbadessa

PONE-D-25-18056R1

PLOS ONE

Dear Dr. Zheng,

I'm pleased to inform you that your manuscript has been deemed suitable for publication in PLOS ONE. Congratulations! Your manuscript is now being handed over to our production team.

At this stage, our production department will prepare your paper for publication. This includes ensuring the following:

* All references, tables, and figures are properly cited

* All relevant supporting information is included in the manuscript submission,

* There are no issues that prevent the paper from being properly typeset

You will receive further instructions from the production team, including instructions on how to review your proof when it is ready. Please keep in mind that we are working through a large volume of accepted articles, so please give us a few days to review your paper and let you know the next and final steps.

Lastly, if your institution or institutions have a press office, please let them know about your upcoming paper now to help maximize its impact. If they'll be preparing press materials, please inform our press team within the next 48 hours. Your manuscript will remain under strict press embargo until 2 pm Eastern Time on the date of publication. For more information, please contact onepress@plos.org.

If we can help with anything else, please email us at customercare@plos.org.

Thank you for submitting your work to PLOS ONE and supporting open access.

Kind regards,

PLOS ONE Editorial Office Staff

on behalf of

Dr. Gianmarco Abbadessa

Academic Editor

PLOS ONE
